# NETosis and lack of DNase activity are key factors in *Echis carinatus* venom-induced tissue destruction

**DOI:** 10.1038/ncomms11361

**Published:** 2016-04-19

**Authors:** Gajanan D. Katkar, Mahalingam S. Sundaram, Somanathapura K. NaveenKumar, Basavarajaiah Swethakumar, Rachana D. Sharma, Manoj Paul, Gopalapura J. Vishalakshi, Sannaningaiah Devaraja, Kesturu S. Girish, Kempaiah Kemparaju

**Affiliations:** 1 Department of Studies in Biochemistry, University of Mysore, Manasagangothri, Mysuru 570006, India; 2 Department of Studies and Research in Biochemistry, Tumkur University, Tumkuru 572103, India

## Abstract

Indian *Echis carinatus* bite causes sustained tissue destruction at the bite site. Neutrophils, the major leukocytes in the early defence process, accumulate at the bite site. Here we show that *E. carinatus* venom induces neutrophil extracellular trap (NET) formation. The NETs block the blood vessels and entrap the venom toxins at the injection site, promoting tissue destruction. The stability of NETs is attributed to the lack of NETs-degrading DNase activity in *E. carinatus* venom. In a mouse tail model, mice co-injected with venom and DNase 1, and neutropenic mice injected with the venom, do not develop NETs, venom accumulation and tissue destruction at the injected site. Strikingly, venom-induced mice tail tissue destruction is also prevented by the subsequent injection of DNase 1. Thus, our study suggests that DNase 1 treatment may have a therapeutic potential for preventing the tissue destruction caused by snake venom.

## Abstract (web summary)

The saw-scaled viper venom causes continued tissue damage that may cause death. Here the authors show that the venom attracts neutrophils to the bite site and induces neutrophil extracellular traps that capture venom components causing tissue damage, which can be prevented by enzymatic DNA degradation.

## Graphical abstract

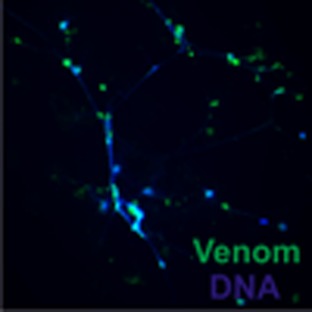

Snakebites affect millions of people worldwide and cause injury, disability and death. Annual epidemiology data have recorded ∼5.5 million bites, including 0.4 million amputations and 0.125 million deaths^1^,^2^,^3^. However, the public health importance of snakebites has been neglected^3^. Thus, in 2009, the World Health Organization categorized snakebite as a ‘Neglected tropical disease'^3^. Snakebite causes both fatal systemic and local toxicities. The local toxicity is characterized by the continued tissue destruction, which predominantly results from viper bites. Although antivenom therapy has saved many lives, it has failed to inhibit viper bite-induced tissue destruction^4^. In addition, studies have demonstrated that Metzincin family matrix-degrading snake venom metalloproteases (SVMPs)^5^ and hyaluronidases (SVHYs) induce local tissue destruction^6^,^7^,^8^; unfortunately, their neutralization by natural and synthetic compounds has failed to reach the clinic^9^,^10^,^11^. This is not due to lack of neutralizing potency of the antivenoms or ineptness of the inhibitors, but rather to the rapid development of local pathology with an unknown cause, which prevents the therapeutic antibodies/inhibitors from accessing the damaged site^1^.

*Echis* species (saw-scaled/carpet vipers) envenomation is well known for producing tissue destruction at the bite site and accounts for the largest number of cases of mortality and morbidity resulting from snakebite in northern Africa and Asia^10^,^12^. *Echis* species venom is rich in SVMPs, which are multidomain haemorrhagic proteases that contain additional cysteine-rich and C-type lectin-like domains^13^,^14^. These additional domains are largely responsible for the recruitment of inflammatory cells that trigger inflammation^14^. Neutrophils are the first-line defence cells in innate immunity, and they infiltrate and accumulate at the bite site^15^; however, their role in tissue destruction remains unknown^16^. These cells quickly respond to foreign agents through phagocytosis and respiratory burst, but when required, they readily die by discharging their decondensed chromatin covered with cytotoxic and antimicrobial agents, known as neutrophil extracellular traps or NETs, in a process-dubbed NETosis^17^,^18^. The defensive role of NETs/extracellular DNA in immobilizing and killing pathogens has been well documented^17^ and is termed as an ancient defence weapon^19^. Paradoxically, NETs also elicit collateral damage because of their associated cytotoxic components^20^,^21^,^22^. Thus, NETs work like a double-edged sword^23^. This led us to focus on and explore the role played by neutrophils in the tissue destruction induced by *E. carinatus* venom. As neutrophils accumulate at the site of venom injection, we hypothesized that the venom triggers NETosis. NETs may play a critical role in the entrapment and accumulation of venom toxins at the bite/injection site, which could be a trigger that accelerates tissue destruction.

Here we demonstrate that *E. carinatus* venom causes formation of NETs, resulting in the accumulation of venom toxins at the injection site and leading to continued tissue degradation. We also show that NETs could be degraded by externally added DNase 1, which could be a possible treatment for this type of snakebite.

## Results

### *E. carinatus* venom stimulates neutrophils to promote NETosis

**Figure 1:**
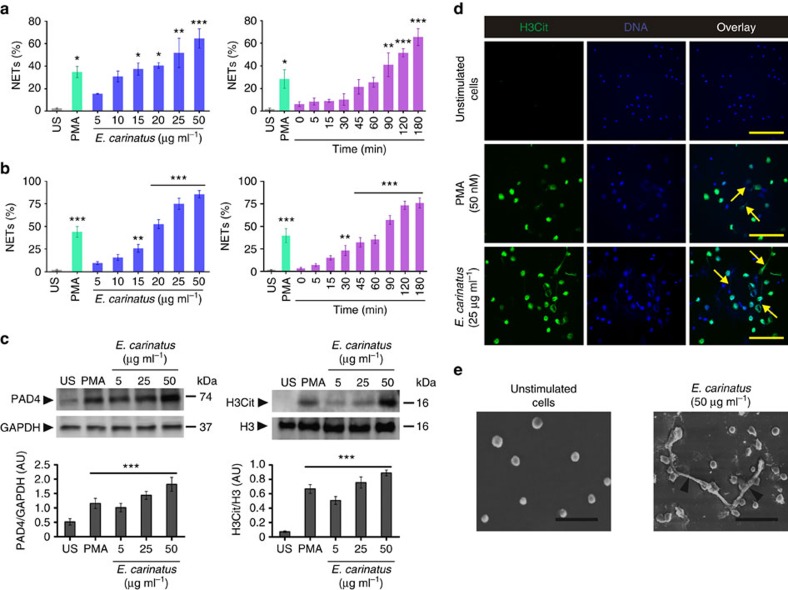
*E. carinatus* venom stimulates *ex vivo* NETosis. *E. carinatus* venom-stimulated NET formation was quantified using (**a**) MPO-DNA capture ELISA and (**b**) Hoechst staining in dose- (left) and time-dependent (right) assays. The results are expressed as the percent increase relative to unstimulated cells (US); mean±s.e.m. (*n*=6). *P<0.05, **P<0.01, ***P<0.001 versus US; one-way analysis of variance (ANOVA), followed by Dunnett's *post-hoc* test. PMA (50 nM) served as a positive control. (**c**) Western blot analysis of PAD4 expression (top, left) and the presence of H3Cit (top, right) in *E. carinatus* venom-treated neutrophils. PAD4 expression was normalized to GAPDH expression (bottom, left), and H3Cit levels were normalized to H3 levels (bottom, right). AU, arbitrary units; H3Cit, citrullinated histone 3; H3, histone 3; US, unstimulated cells. The data are presented as mean±s.e.m. (*n*=4). ***P<0.001 versus US; one-way ANOVA, followed by Dunnett's *post-hoc* test. PMA (50 nM) served as a positive control. The PVDF membranes were cut based on molecular weight of respective protein using protein molecular weight marker and then probed with respective antibodies. (**d**) Representative immunofluorescence images of neutrophils/NETs. The neutrophils were exposed to *E. carinatus* venom (25 μg ml^−1^) for 2.5 h at 37 °C. Yellow arrows indicate NETs. (*n*=4) Scale bars, 100 μm. PMA (50 nM) served as a positive control. (**e**) Scanning electron microscopy images showing unstimulated neutrophils (left) and *E. carinatus* venom-stimulated neutrophils, which displayed NETs with thick bundles of fibres (black arrowheads; right). (*n*=4) Scale bars, 30 μm.

**Figure 2:**
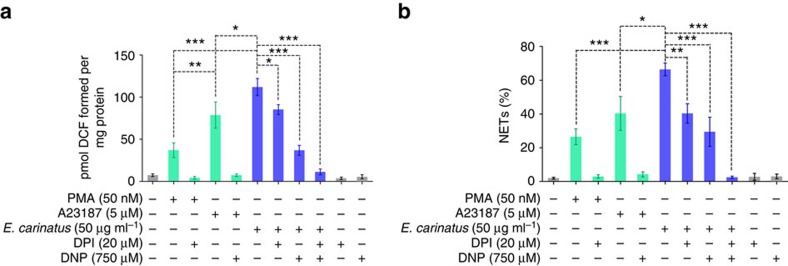
*E. carinatus* venom-mediated NETosis is both NOX-dependent and NOX-independent. (**a**) Represents the level of ROS in neutrophils incubated with PMA, A23187 or *E. carinatus* venom in the presence or absence of DPI (20 μM) or DNP (750 μM) or both DPI (20 μM), DNP (750 μM). The data are presented as mean±s.e.m. (*n*=4). *P<0.05, **P<0.01, ***P<0.001; one-way analysis of variance followed by Bonferroni *post-hoc* test. (**b**) Represents NET release measured using MPO-DNA capture ELISA in neutrophils incubated with PMA, A23187 or *E. carinatus* venom in the presence or absence of DPI (20 μM) or DNP (750 μM) or both DPI (20 μM) DNP (750 μM). The data are presented as mean±s.e.m. (*n*=4). *P<0.05, **P<0.01, ***P<0.001; one-way ANOVA, followed by Bonferroni *post-hoc* test.

We tested whether *E. carinatus* venom could induce NETosis in human neutrophils. The venom induced NET formation in both dose- and time-dependent manner, and the NETs were quantified using myeloperoxidase-DNA (MPO-DNA) capture ELISA (Fig. 1a, left and right) and Hoechst staining (Fig. 1b, left and right) assays. The venom-treated neutrophils showed a dose-dependent increase in the expression of the peptidylarginine deiminase 4 (PAD4) enzyme (Fig. 1c, left), and this paralleled with the formation of citrullinated histone H3 (H3Cit; Fig. 1c, right) in western blot studies. Furthermore, the immunocytochemistry study revealed that H3Cit and the extracellular DNA co-localize (Fig. 1d). The quantification of the H3Cit-positive neutrophils and their extruded DNA indicated that they were significantly increased compared with unstimulated neutrophils (Supplementary Fig. 1a,b). Phorbol 12-myristate 13-acetate (PMA)-treated neutrophils served as positive control. Scanning electron microscope analysis confirmed the NETosis, where thick bundles of chromatin fibres, NETs, emerging from and connecting different neutrophils were conspicuously visible compared with the intact, unstimulated neutrophils (Fig. 1e). We next examined the *E. carinatus* venom-induced dose-dependent reactive oxygen species (ROS) production in neutrophils (Supplementary Fig. 2). The venom-induced ROS production was decreased when neutrophils were pre-incubated with diphenyleneiodonium chloride (DPI) or dinitrophenol (DNP) or together (Fig. 2a). However, DNP decreased the ROS production more significantly than DPI, whereas in combination the effect was found to be additive (Fig. 2a). Similarly, the trend was paralleled with the quantity of NETs formation (Fig. 2b).

**Figure 3:**
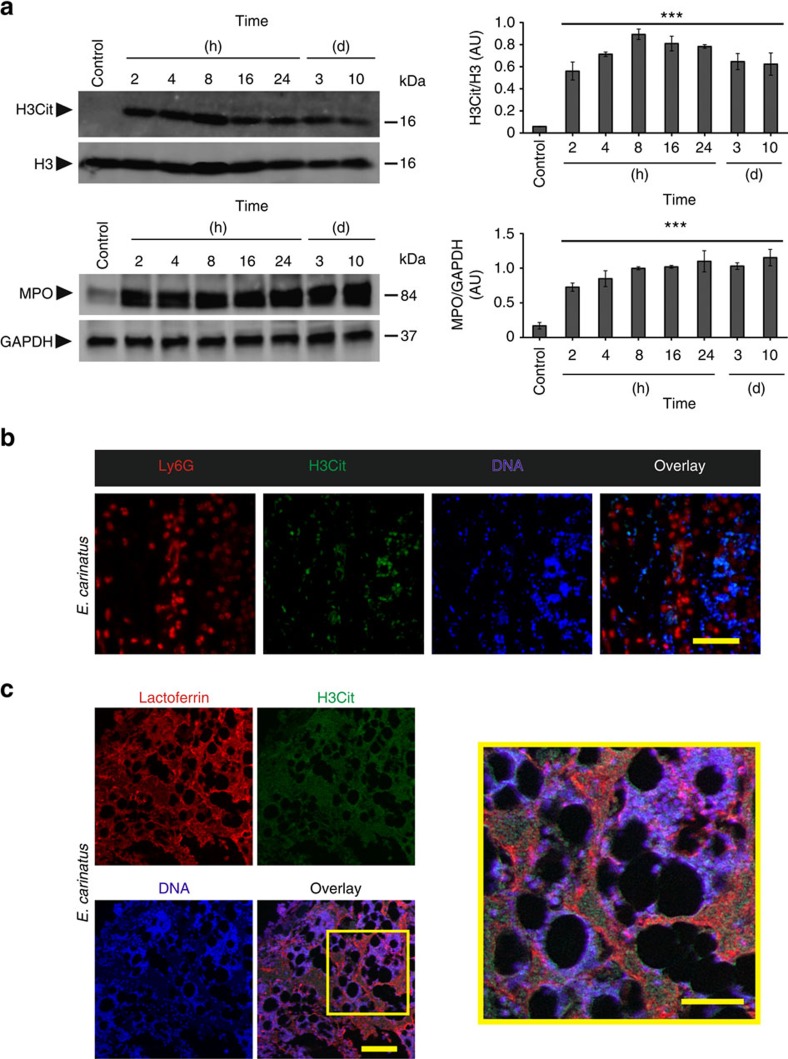
*E. carinatus* venom stimulates *in vivo* NETosis. (**a**) Representative western blot of the time course of H3Cit and MPO appearance in *E. carinatus* venom (LD_50_)-injected mouse tail tissue (left) and quantification of the H3Cit levels compared with the H3 levels (top, right) and MPO level compared to GAPDH (bottom, right). AU, arbitrary units; H3Cit, citrullinated histone 3; H3, histone 3; MPO, myeloperoxidase. The data are presented as mean±s.e.m; Student's *t*-test, ***P<0.001 versus control; *n*=4 for the control, 2, 4, 8 and 16 h samples; *n*=5 for 24 h, day 3 and day 10 samples. The PVDF membranes were cut based on molecular weight of respective protein using protein molecular weight marker and then probed with respective antibodies. (**b**) Representative immunofluorescence images of mouse tail tissue 8 h after *E. carinatus* venom (LD_50_) injection, focused beneath the epithelial layer. Scale bar, 100 μm (*n*=4). (**c**) Representative confocal image of *E. carinatus* venom (LD_50_)-injected mouse tail tissues focused beneath the epithelial layer. The area enclosed by the yellow box is magnified and shown on the right. Scale bars, 100 μm (left), 50 μm (right); *n*=3.

In the *in vivo* experiment, mice that were given injections of *E. carinatus* venom (lethal dose_50_ (LD_50_) is the amount of venom that causes 50% mortality) into the tail were observed for 15 days. The appearance of haemorrhage and tissue destruction at the site of injection was noticed ∼1 h after venom injection. The tail exhibited severe haemorrhage and tissue destruction 8 h after venom injection compared with the control, which received phosphate-buffered saline (PBS; Supplementary Fig. 3a). The venom-injected tails were monitored and visually scored for injury for 15 days. The injured tails detached between days 10 and 15 (Supplementary Fig. 3b). Western blotting of the venom-injected tail tissue homogenates revealed increased levels of H3Cit and MPO that reached a maximum at 8 h and persisted even after the tail became necrotic (day 3 and onwards) and detached (day 10 and onwards; Fig. 3a). Tail tissue sections taken 8 h after venom injection showed the destruction and loss of integrity of the dermis and hypodermis, along with extruded DNA in the haematoxylin and eosin (H&E) staining compared with the PBS-injected control tail tissue sections (Supplementary Fig. 3c). Furthermore, immunofluorescence images of the tail tissue sections were positive for Ly6G, H3Cit and extracellular DNA, indicating that NETosis occurred in the venom-injected tail tissue (Fig. 3b and Supplementary Fig. 3d). A confocal microscopy study confirmed the existence of NETs in the venom-injected tail tissues, as evidenced by the co-localization of lactoferrin, H3Cit and extracellular DNA (Fig. 3c and Supplementary Fig. 4). However, H3Cit antibody also showed some nonspecific binding in mice tail tissues (Supplementary Fig. 4).

### NETs block blood vessels and capture *E. carinatus* venom

**Figure 4:**
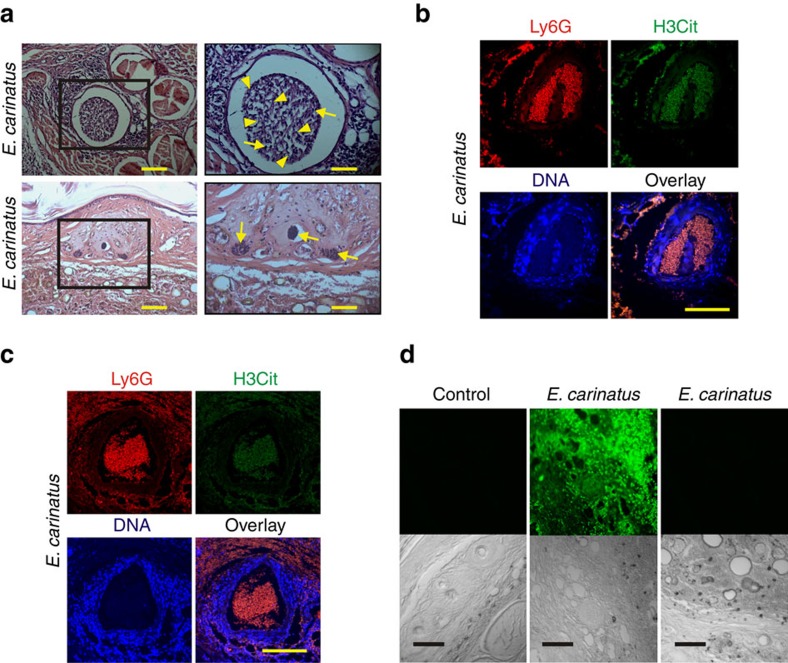
NETs block blood vessels, leading to accumulation of *E. carinatus* venom. (**a**) Images of H&E-stained mouse tail tissue 8 h after *E. carinatus* venom (LD_50_) injection show clot formation in the mouse tail vein (top, left) and blocked blood capillaries (bottom, left). The respective enlarged images are shown on the right. Scale bars, 50 μm (left), 100 μm (right). Yellow arrowheads indicate NETs and yellow arrows indicate neutrophils. Immunofluorescence image of the mouse (*n*=4). (**b**) tail artery and (**c**) vein 8 h after *E. carinatus* venom (LD_50_) injection. Scale bars, 100 μm (*n*=4). (**d**) Immunofluorescence images show the accumulation of venom in *E. carinatus* venom (LD_50_)-injected mouse tail tissue section (top row, middle), section with a secondary antibody control (top row, right) and section of PBS-injected tail tissue, which served as a control (top, left). The corresponding differential interference contrast (DIC) images of the respective tissues are also shown (bottom row). Scale bars, 100 μm (*n*=4).

H&E-stained tail tissue sections obtained 8 h after venom injection showed that the veins and capillaries (Fig. 4a) were blocked owing to the accumulation of NETs. Immunohistochemistry of the tail tissue sections further confirmed the accumulation and blockage of artery (Fig. 4b) and vein (Fig. 4c) by the NETs, as evidenced by the co-localization of Ly6G, H3Cit and extracellular DNA. Furthermore, *E. carinatus* venom accumulated in these tissue sections, even at 8 h after the venom injection, as demonstrated using a rabbit polyclonal antivenom raised against *E. carinatus* venom (Fig. 4d).

**Figure 5:**
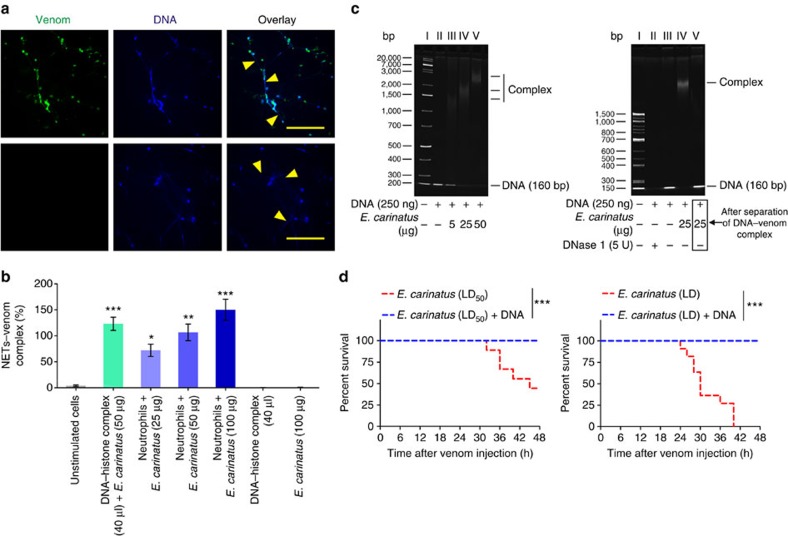
NETs capture *E. carinatus* venom toxins. (**a**) Representative immunofluorescence image of neutrophils exposed to *E. carinatus* venom (50 μg ml^−1^) for 2.5 h; the venom was detected using rabbit polyclonal antibody against *E. carinatus* venom followed by an AlexaFluor 488-conjugated goat anti-rabbit antibody along with DNA stained by Hoechst stain (top row). The AlexaFluor 488-conjugated goat anti-rabbit secondary antibody control (bottom row). Yellow arrow heads indicate NETs. Scale bars, 100 μm (*n*=4). (**b**) The NET–venom complex was quantified using the DNA–venom capture ELISA assay. The results are expressed as a percent increase with respect to the unstimulated cells. The data are presented as mean±s.e.m. (*n*=4). *P<0.05, **P<0.01, ***P<0.001 versus the unstimulated cells; one-way analysis of variance, followed by Dunnett's *post-hoc* test. (**c**) Native PAGE (7.5%) showing the interaction between DNA and *E. carinatus* venom (left), as demonstrated by the dose-dependent retardation of the bands. Marker (lane I), 250 ng DNA (lane II), 250 ng DNA+5 μg *E. carinatus* venom (lane III), 250 ng DNA+25 μg *E. carinatus* venom (lane IV) and 250 ng DNA+50 μg *E. carinatus* venom (lane V). Recovery of DNA from the DNA–venom complex (right). Marker (lane I), 250 ng DNA+5 U DNase 1 (lane II), 250 ng DNA (lane III), 250 ng DNA+25 μg *E. carinatus* venom (lane IV) and recovered DNA from DNA+*E. carinatus* venom complex (lane V); *n*=3. (**d**) Kaplan–Meier survival curves show that the lethal potency of the *E. carinatus* venom is inhibited when it is incubated with DNA: *E. carinatus* venom, LD_50_ (red line, left) and LD (red line, right), and *E. carinatus* venom incubated with DNA for 10 min at 37 °C (blue line, both left and right); *n*=10. ***P<0.001. Log-rank test.

**Figure 6:**
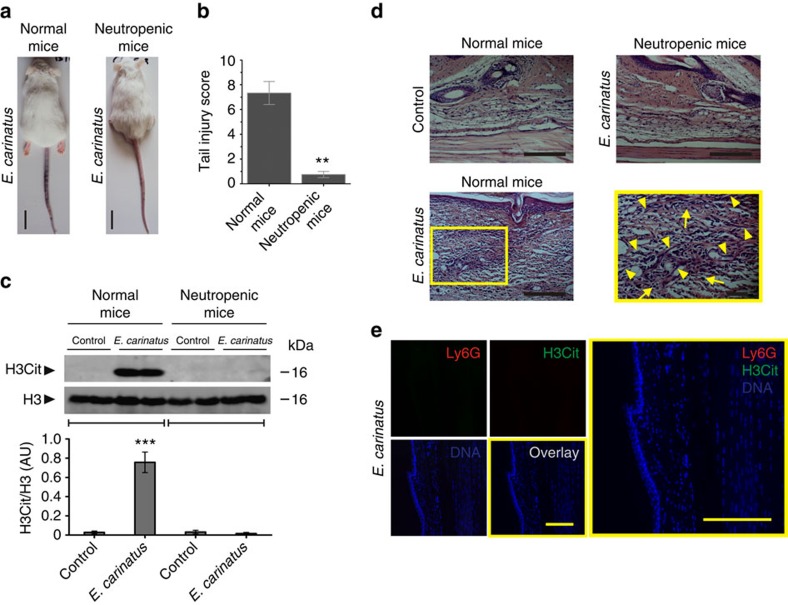
*E. carinatus* venom does not induce local tissue damage or NETosis in neutropenic mice. (**a**) Representative photographs of mice 8 h after *E. carinatus* venom (LD_50_) injection in the tail show intense venom-induced wound in the tail of a normal mouse (left), but no haemorrhage in a neutropenic mouse (right). Scale bars, 2 cm. (*n*=10). (**b**) The corresponding tail injury score 8 h after the *E. carinatus* venom (LD_50_) injection is shown in a bar graph. The data are presented as mean±s.e.m. *n*=6 venom-injected normal mice; *n*=10 venom-injected neutropenic mice. **P<0.01; Student's *t*-test. (**c**) Western blot analysis of the appearance of H3Cit (top) in tail tissue homogenates taken from normal and neutropenic mice 8 h after *E. carinatus* venom (LD_50_) injection. The quantification of H3Cit levels compared with H3 levels is shown (bottom). AU, arbitrary units; H3Cit, citrullinated histone 3; H3, histone 3. The data are presented as mean±s.e.m. (*n*=4). ***P<0.001 versus the normal control mice; one-way analysis of variance, followed by Dunnett's *post-hoc* test. The PVDF membranes were cut based on molecular weight of respective protein using protein molecular weight marker and then probed with respective antibodies. (**d**) H&E-stained tail tissue sections from neutropenic (top right) and normal mice (bottom left) injected with *E. carinatus* venom (LD_50_); the yellow portion is enlarged and shown on the right. Tissue from PBS-injected normal mice is also shown (top, left). Scale bars, 100 μm. (*n*=4). (**e**) Representative immunofluorescence images of neutropenic mouse tail tissue 8 h after *E. carinatus* venom (LD_50_) injection, focused beneath the epithelial layer. Scale bars, 100 μm (*n*=4).

Immunocytochemistry revealed that the NETs captured the venom, as evidenced by the co-localization of the venom toxins and NETs (Fig. 5a). The DNA–venom capture ELISA technique was employed to demonstrate the interaction between the NETs and the *E. carinatus* venom. The formation of the NET–venom complex increased with the increasing amounts of venom (Fig. 5b). The interaction between the DNA and the venom was further analysed using native polyacrylamide gel electrophoresis (PAGE), where the addition of venom to DNA retarded DNA mobility in a dose-dependent manner (Fig. 5c). The DNA–venom complex was dissociated using a DNA isolation protocol and analysed by native PAGE (Fig. 5c). Then, the DNA–venom complex was studied for its lethal potency using independent groups of mice that received the DNA–venom complex prepared using an LD_50_ or LD dose of the venom (venom/DNA: 1:1, w/w; Fig. 5d). Neither group exhibited mortality, while the groups that received LD_50_ and LD of venom alone exhibited 50% and 100% mortality, respectively. In contrast, *E. carinatus* venom failed to induce tail tissue damage in neutropenic mice (Fig. 6a). The tail injury score was decreased by approximately eightfold in neutropenic mice compared with the venom-injected control mice (Fig. 6b). Western blot analysis of tail tissue homogenates from the venom-injected neutropenic mice showed no sign of H3Cit signals (Fig. 6c). H&E staining of tail tissue sections from the venom-injected neutropenic mice showed an absence of neutrophils and, hence, extracellular DNA compared with the venom-injected normal mice (Fig. 6d). Immunohistochemistry further confirmed the lack of neutrophils and NETs, as the sections were negative for Ly6G and H3Cit (Fig. 6e). Whereas the group of neutropenic mice that received the LD_50_ dose of venom exhibited 100% mortality (Supplementary Fig. 5). As DNA is the backbone of NETs, the *E. carinatus* venom-induced stable NETosis was our impetus to test the DNase activity of the venom. We found that the venom failed to degrade herring sperm DNA, even after exhaustive incubation for 24 h (Supplementary Fig. 6).

### DNase 1 prevents *E. carinatus* venom-induced NETosis

**Figure 7:**
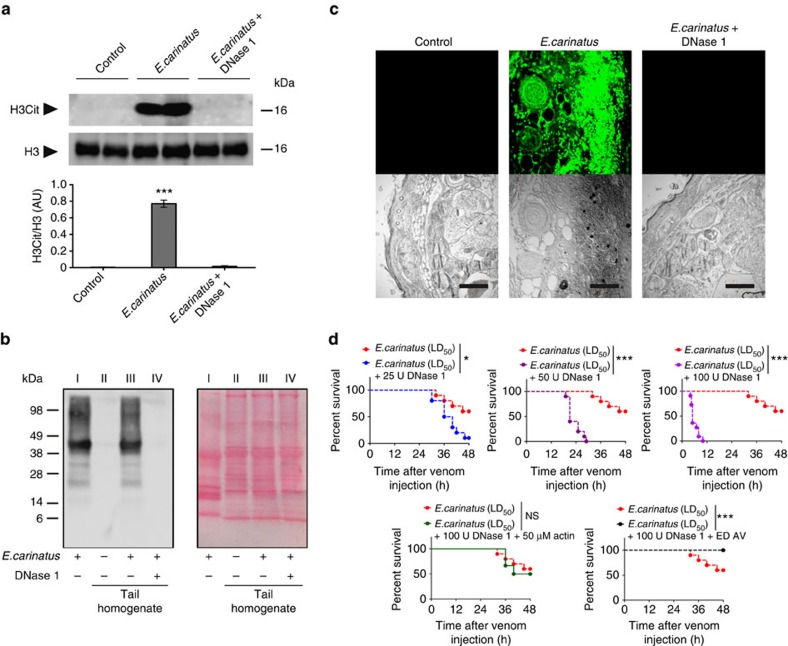
Co-injection with DNase 1 prevents *E. carinatus* venom-induced tissue destruction. (**a**) Western blot analysis of the appearance of H3Cit (top) in tail tissue homogenates taken 8 h after *E. carinatus* venom (LD_50_) injection in the presence or absence of DNase 1. Quantification of H3Cit levels compared with H3 levels is shown (bottom). AU, arbitrary units; H3Cit, citrullinated histone 3; H3, histone 3. The data are presented as mean±s.e.m. (*n*=4). ***P<0.001 versus control mice; one-way analysis of variance, followed by Dunnett's *post-hoc* test. The PVDF membranes were cut based on molecular weight of respective protein using protein molecular weight marker and then probed with respective antibodies. (**b**) Western blot analysis (left) of the appearance of *E. carinatus* venom in tail tissue homogenates taken 8 h after venom (LD_50_) injection in the presence or absence of DNase 1. *E. carinatus* venom (20 μg) served as a positive control. The image of the corresponding Ponceau-stained PVDF membrane (right) shows *E. carinatus* venom, 20 μg (lane I), and equal protein loading of the tail tissue homogenates (lane II–IV); *n*=3. (**c**) Immunofluorescence images showing the accumulation of venom toxins from *E. carinatus* venom (LD_50_)-injected mouse tail tissues sections in the absence (top row, middle) or presence of DNase 1 (100 U; top row, right). PBS-injected tail tissue served as a control (top row, left). The corresponding DIC images of the respective tissues are also shown (bottom row). Scale bars, 100 μm (*n*=3). (**d**) All the experiments in this group were performed simultaneously but the data are divided into five graphs (top three and bottom two) for clarity. Kaplan–Meier survival curves: *E. carinatus* venom, LD_50_ (red line) in all the graphs; co-injection of *E. carinatus* venom (LD_50_) with 25 U DNase 1 (blue line), 50 U DNase 1 (green line), 100 U DNase 1 (violet line), 100 U DNase 1 pre-incubated with 50 μM actin (grey line) and 100 U DNase 1 followed by ED AV (black line). ED AV, effective dose of antivenom. (*n*=10). *P<0.05, ***P<0.001 and NS, non-significant between groups using the Log-rank test. See Supplementary Fig. 5 for the effect of DNase 1 on the lethal potency of *E. carinatus* venom (LD).

In *in vivo* studies, co-injection of DNase 1 prevented the *E. carinatus* venom-induced tail tissue damage in a dose-dependent manner (Supplementary Fig. 7a). However, the tissue-damaging property of the venom was not altered by co-injection with actin pre-treated DNase 1 (Supplementary Fig. 7a). The western blot study revealed that H3Cit (Fig. 7a) and venom (Fig. 7b) were not present in tail tissue homogenates from mice injected with both venom and DNase 1, whereas the homogenates from mice injected with venom alone contained both H3Cit (Fig. 7a) and venom (Fig. 7b). Venom accumulation was further demonstrated using immunofluorescence of the tail tissue sections (Fig. 7c). Although the mice injected with both venom and DNase 1 did not show any signs of local tissue damage, interestingly, the mice died much faster than if they were injected with venom alone (Fig. 7d and Supplementary Fig. 7b).

**Figure 8:**
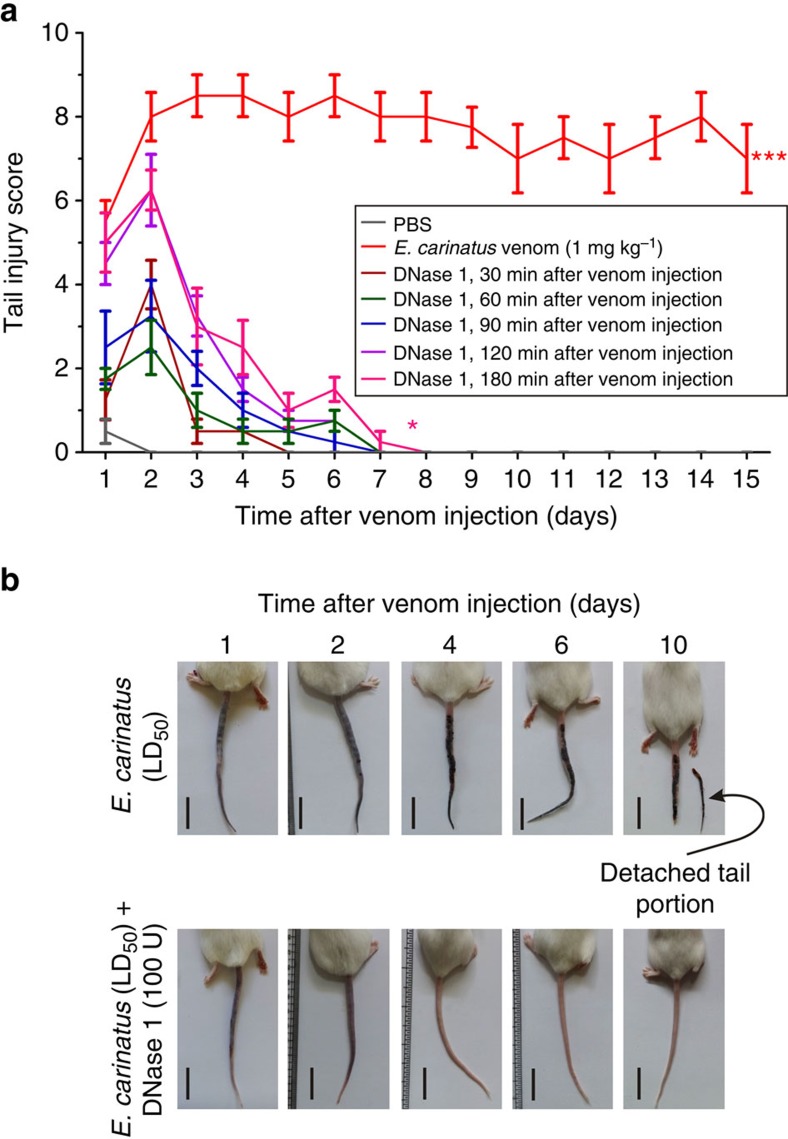
DNase 1 treatment prevents *E. carinatus* venom-induced tissue destruction in the challenge study. (**a**) The graph represents the continued high injury score in *E. carinatus* venom (LD_50_)-injected mouse tails (red line), whereas the administration of 100 U DNase 1 at various times (30–180 min post-venom injection) decreased the tail injury score. The data are presented as mean±s.e.m. (*n*=10). *P<0.05, ***P<0.001 versus PBS injected control mice; one-way analysis of variance, followed by Dunnett's *post-hoc* test. (**b**) Representative photographs of mice taken on different days after injection. The mice were injected with *E. carinatus* venom (LD_50_; top row) or co-injected with *E. carinatus* venom (LD_50_) and DNase 1 (100 U; bottom row); the mice in the latter group recovered and normal tail morphology was restored on day 4 onwards (bottom, third). Scale bars, 2 cm (*n*=10).

In the challenge study, the tail injury score (Fig. 8a) and photographs of representative mice (Fig. 8b) revealed that when DNase 1 was injected at different time intervals after the venom (LD_50_) injection (30–180 min), the mice tails showed initial signs of haemorrhage on days 1 and 2, and recovered on days 5–7, exhibiting normal tail morphology without an increase in lethality. Conversely, in the absence of the DNase 1 treatment, intense haemorrhage and wound formation led to the detachment of the affected tail portion between days 10 and 15 (Fig. 8a,b). Moreover, when serum was incubated with *E. carinatus* venom, the serum DNase activity was not inhibited, as evidenced by the nearly equal zone of clearance in serum alone and in serum incubated with the increasing doses of *E. carinatus* venom (Supplementary Fig. 8).

**Figure 9:**
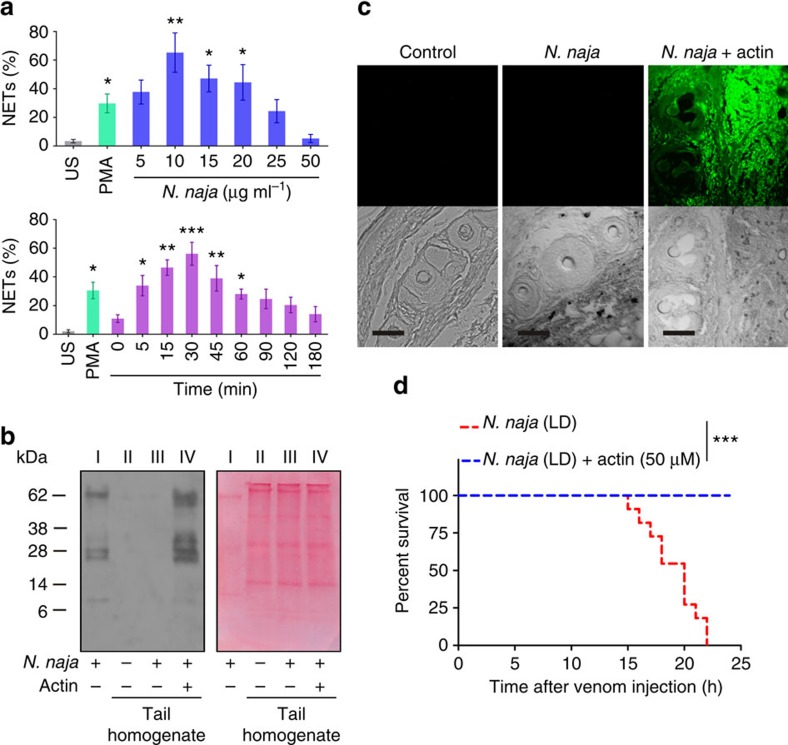
DNase activity of *N. naja* venom is essential for degrading NETs and increasing its lethal potency. (**a**) *N. naja* venom-stimulated NET formation was quantified using MPO-DNA capture ELISA in both dose- (top) and time-dependent (bottom) assays. US, unstimulated cells. The results are expressed as the percent increase relative to the US; mean±s.e.m. (*n*=6). *P<0.05, **P<0.01, ***P<0.001 versus the US; one-way analysis of variance, followed by Dunnett's *post-hoc* test. PMA (50 nM) served as a positive control. (**b**) Western blot analysis (left) of the appearance of *N. naja* venom in tail tissue homogenates taken 8 h after venom (LD) injection in the presence or absence of actin (50 μM). *N. naja* venom (20 μg) served as a positive control. The image of the corresponding Ponceau-stained PVDF membrane (right) shows *N. naja* venom, 20 μg (lane I) and equal protein loading of tail tissue homogenates (lane II–IV); *n*=3. (**c**) Representative immunofluorescence images were captured 8 h after *N. naja* venom (LD) injection in mouse tails and did not show an accumulation of venom (top row, middle), similar to the PBS-injected control tissue (left). However, *N. naja* venom accumulated when the venom (LD) was pre-treated with 50 μM actin before injection. The corresponding DIC images of respective tissues are shown (bottom). Scale bars, 100 μm (*n*=3). (**d**) Kaplan–Meier survival curves after injections of *N. naja* venom (LD, red line) or *N. naja* venom (LD) pre-incubated with 50 μM actin (blue line); *n*=10. ***P<0.001; Log-rank test.

To determine whether venom from another snake species, *N. naja*, induces NETosis, we used MPO-DNA capture ELISA assay. We did observe NETosis at lower doses of the venom, but it was not observed at higher doses or with prolonged incubation periods (Fig. 9a). Western blot study using a rabbit polyclonal antivenom for *N. naja* venom showed that the venom did not accumulate at the injection site (Fig. 9b). This was further confirmed by the immunohistochemistry study, in which tail tissue sections from venom-injected mice did not show an accumulation of venom at the injection site. However, in mice injected with actin pre-treated *N. naja* venom, the tail tissue sections exhibited an accumulation of the venom (Fig. 9b,c). Furthermore, the actin pre-treated *N. naja* venom failed to induce lethal toxicity, as all of the experimental mice survived (Fig. 9d). Finally, when tested for DNase activity, the *N. naja* venom dose-dependently hydrolysed calf thymus DNA, whereas actin pre-treated venom failed to hydrolyse DNA (Supplementary Fig. 9).

## Discussion

*E. carinatus* induces intense tissue destruction at the bite site, with symptoms such as oedema, haemorrhage and tissue destruction^10^,^24^. Although neutrophil infiltration has been demonstrated at the site of a viper bite/venom injection^15^, it has not been thoroughly investigated^8^. This study deciphers the cellular mechanism of tissue destruction by the *E. carinatus* venom and suggested a possible therapeutic approach for snakebite management. We show that the venom-stimulated neutrophils undergo NETosis and that the resulting NETs block the blood vessels to prevent the venom from entering the circulation, resulting in venom accumulation and tissue destruction at the injection site. This effect was reversed by the administration of DNase 1.

Neutrophils are the first-line defence cells and effectively capture pathogens by NETosis^17^. NETosis occurs via both the NOX-dependent and NOX-independent pathways^25^. Our study suggests that *E. carinatus* venom stimulated the neutrophils to undergo NETosis both *in vitro* and *in vivo*. *E. carinatus* venom-stimulated ROS production and NETosis were significantly decreased in the presence of DPI (NOX inhibitor) and DNP (uncoupler of oxidative phosphorylation and electron transport) indicating the induction of both the NOX-dependent and NOX-independent pathways. Induction of both pathways is likely due to the presence of various toxins in *E. carinatus* venom that affects several cellular pathways. Some strains of *Staphylococcus aureus* and lipid mediator hepoxilin A3 induce NETosis through both the NOX-dependent and NOX-independent patways^26^,^27^,^28^. Several recent studies have demonstrated that *E. carinatus* venom induced oxidative stress in both *ex vivo* and *in vivo* experiments^10^,^29^. NETosis is marked by increased PAD4 expression, which triggers chromatin de-condensation through deimination of arginine in H3 to form citrulline (H3Cit)^30^,^31^. Thus, the results of the western blot shows increased expression of PAD4 and the appearance of H3Cit in human neutrophils support the hypothesis that the *E. carinatus* venom induces NETosis. The rapid increase in PAD4 levels in neutrophils could be due to *E. carinatus* venom-stimulated NOX-independent pathway^25^,^32^. We show that *E. carinatus* venom does not contain bacteria, which rules out the role of bacterial contamination in driving neutrophils into NETosis (Supplementary Fig. 10). The detection of NETosis markers, such as Ly6G, lactoferrin and H3Cit, in venom-injected tail tissue sections confirmed NETosis *in vivo*.

Furthermore, the sections exhibited blood vessel obstruction due to the accumulation of blood clots, including NETs. *E. carinatus* venom is a pro-coagulant^33^ and promotes fibrin clot formation^10^,^34^. Moreover, NETs also promote fibrin clot formation^35^,^36^. NET-associated clots are more resistant to mechanical forces and enzymatic digestion^35^,^37^; thus, the blood vessels remained blocked, even after 8 h of venom injection, despite the fibri(noge)nolytic activity of *E. carinatus* venom^10^,^24^. However, further detailed investigation is warranted to understand the interactions between NETs and fibrin clots. The blockage of blood vessels prevents the easy entry of the venom into the circulation and causes its accumulation at the injection site. The accumulated venom is a cocktail of several enzymatic and non-enzymatic toxins, predominantly the extracellular matrix (ECM)-degrading haemorrhagic SVMPs and SVHYs. SVMPs degrade structural proteins, including collagens, laminin and fibronectin molecules^5^,^38^,^39^, whereas SVHYs degrade the structural glycosaminoglycan hyaluronic acid^6^,^8^,^40^. Thus, the loss of ECM integrity destabilizes the tissues and the basement membrane of blood vessels. In addition, NET-associated histones can damage endothelial cells^20^,^21^,^23^. Furthermore, NET-associated neutrophil elastase is a highly stable broad-spectrum protease that can cause further ECM damage^20^,^22^,^23^,^30^. In a recent study, NETosis was shown to cause delayed wound healing in diabetic patients^32^. Therefore, *E. carinatus* venom-induced NETosis not only restricts the venom to the bite site and promotes tissue destruction but also blocks the blood flow to the tissues and cells that are distal to the bite site. Increased NET formation or impaired NET clearance is detrimental, as demonstrated in chronic inflammatory disorders, including vasculitis, psoriasis, preeclampsia, atherosclerosis, systemic lupus erythematosus and gout^23^,^41^,^42^,^43^.

Chromatin is an ancient defence weapon of the innate immune system^19^; it is known to form a complex net that enmeshes and kills bacteria, fungi, viruses and other parasites^17^. We demonstrate that the venom-induced NETs capture venom toxins. The negative charge in the NETs^44^ could sequester the positively charged venom toxins such as basic PLA_2_s, 3FTx and SVMPs^45^,^46^,^47^,^48^. The successful recovery of DNA from the DNA–venom complex suggests that the interaction between the DNA and venom toxins was non-covalent. Surprisingly, the lethality studies showed that the lethal potency of the venom was reduced when it formed complex with the DNA. This was attributed to the sequestration and inhibition of the positively charged venom toxins by the NETs/DNA. We reasoned that the depletion of the neutrophils and, hence, the NETs would result in decreased venom-induced tissue destruction and increased lethal potency. Indeed, in neutropenic mice, the venom did not induce haemorrhage and tissue destruction at the injection site, but caused 100% mortality even at the LD_50_ dose of venom. Of note, and as discussed above, although *E. carinatus* venom-induced NETosis is detrimental, it is an adaptive immune response that restricts the entry of *E. carinatus* venom into the circulation and protect from its lethal systemic toxicity.

The stability of the NETs is due to the lack of DNase activity in the *E. carinatus* venom and the inability of serum DNase to clear the large accumulated NETs at the injection site. We ruled out the possible inhibition of serum DNase by the *E. carinatus* venom. Thus, we tested whether DNase 1 could clear the NETs. As anticipated, co-injection of venom and DNase 1 prevented haemorrhage and local tissue damage, but also increased the lethal potency of the venom by improving penetration of the venom into the circulation. This effect was neutralized by antivenom administration. Surprisingly, in the challenge study, all the mice survived when DNase 1 was administered 30–180 min after venom injection. This mitigation of the lethal potency of the venom could be due to the formation of the NETs/DNA–venom complex. We postulate that venom initially stimulates NETosis of the accumulated neutrophils. Eventually, the negatively charged NETs/DNA fibres form complexes with the positively charged venom toxins such as basic PLA_2_s, 3FTxs and SVMPs and entrap them. Further, it is likely that the negatively charged NETs/DNA would chelate metal ions^49^ such as Zn^2+^ and Ca^2+^ thereby inhibiting SVMPs and venom PLA_2_s, respectively^46^,^48^,^50^. Although the NETs/DNA fibres are degraded by the administered DNase 1, it is possible that the small DNA fragments remained in complex with the venom toxins keep them inactive. However, further studies are required to delineate the precise molecular mechanism(s) at play. NET clearance is essential for the wound-healing process^32^. Our present findings are complemented by recent studies showing that DNase 1 treatment accelerated wound resolution in diabetic and wild-type mice^32^ and increased NET clearance by macrophages *in vitro*^51^. In contrast to the *E. carinatus* venom, which is known for its ability to induce haemorrhage and local tissue destruction, the *N. naja* venom lacks these effects^52^. This is most likely due to the fact that the *N. naja* venom contains high levels of DNase activity and this degrades NETs and prevents the venom from accumulating at the injection site, as the inhibition of DNase activity not only resulted in venom accumulation at the injection site but also abolished the venom's lethality. In addition, despite the venom accumulation at the injection site, there was no local tissue destruction. This is likely due to the fact that *N. naja* venom lacks ECM-degrading haemorrhagic SVMPs^52^.

In summary, the results indicate that *E. carinatus* venom induces the formation of stable NETs owing to its lack of DNase activity. These NETs trap the venom toxins and thereby promote tissue destruction at the injection/bite site. The dense NETs and the blood clots formed in the damaged tissues and capillaries hinder the free flow of blood and prevent the antivenom from reaching the damaged site. This study presents (i) the role of NETosis in *E. carinatus* venom-induced tissue destruction, (ii) a convenient mouse tail model to study viper venom-induced tissue destruction, (iii) the role of DNase activity in venom toxicity and (iv) provides a lead for designing new strategies for the possible use of DNase 1 in the management of *E. carinatus* venom-induced tissue destruction. This study could also drive future studies to understand and uncover the molecular mechanisms associated with other venomous bites that cause similar pathological conditions. However, further detailed and systematic investigations in human subjects are needed to validate these findings before they are implemented in the clinic.

## Methods

### Animals

Adult Swiss albino mice (8- to 10-week-old male or female) weighing 20–25 g were obtained from the Central Animal House Facility, Department of Zoology, University of Mysore, Mysuru, India. New Zealand albino female rabbits (6-month-old) weighing 1.5–2 kg were obtained from the Department of Livestock Production and Management, Veterinary College, Bengaluru, India. The animal experiments were approved by the Institutional Animal Ethical Committee, University of Mysore, Mysuru (Approval numbers: UOM/IAEC/20/2012 and UOM/IAEC/09/2013). During all experiments, animal care and handling were in accordance with the guidelines of the Committee for the Purpose of Control and Supervision of Experiments on Animals (CPCSEA).

### Humans

Human blood was drawn from the antecubital veins of healthy adult volunteers who provided written informed consent, as per the guidelines of the Institutional Human Ethical Committee, University of Mysore, Mysuru. All the experiments were approved by the Institutional Human Ethical Committee, University of Mysore, Mysuru (Approval number: IHEC-UOM No. 47Res/2014–15) and conducted in accordance with the ethical guidelines.

### Reagents

The *E. carinatus* and *N. naja* venoms were purchased from Irula Co-operative Society Ltd., Chennai, India. The calf thymus DNA, DMEM, Histopaque-1077, Ponceau stain, actin from bovine muscle, protease inhibitor cocktail, protein A agarose, dextran (molecular weight∼100 kDa), PMA, bovine serum albumin, human serum albumin (HSA), calcium ionophore (A23187), 2′,7′-dichlorofluorescein diacetate (DCFDA), DPI, DNP, Cell Death Detection ELISA^PLUS^ (Version 14, Roche Diagnostics), and Freund's complete and incomplete adjuvants were procured from Sigma Chemicals. DNase 1 was purchased from Boehringer Ingelheim. The anti-histone H3 antibody (Cat. no. SC-10809) was obtained from Santa Cruz Biotechnology, Inc. Hoechst 33342 (Cat. no. H3570) was obtained from Life Technologies. AlexaFluor 488-conjugated AffiniPure Goat Anti-Rabbit IgG (H+L) (Cat. no. 111-545-003), AlexaFluor 647-conjugated AffiniPure Goat Anti-mouse IgG (H+L) (Cat. no. 115-605-003) were purchased from Jackson Immuno Research Laboratories, Inc. The AlexaFluor 647-conjugated anti-mouse Ly 6G antibody (clone 1A8; Cat. no. 127609) was obtained from BioLegend. The rabbit polyclonal anti-histone H3 (citrulline R2+R8+R17; H3Cit; Cat. no. ab5103), mouse monoclonal anti-myeloperoxidase (anti-MPO; 2C7; Cat. no. ab25989), anti-lactoferrin antibody (2B8; Cat. no. ab10110) and anti-PAD4 (4H5; Cat. no. ab128086) antibodies were obtained from Abcam. The DreamTaq Green DNA Polymerase kit (Cat. no. EP0711) was obtained from Thermo Scientific. Endoxan-*N*-(cyclophosphamide injection) was obtained from Baxter Oncology Products. The mouse anti-GAPDH mAb (6C5; Cat. no. CB1001) was purchased from Calbiochem. The microwell plates were obtained from Thermo Fisher Scientific.

### Human neutrophil isolation

The human neutrophils were isolated from the blood of healthy volunteers^44^. The blood was collected and mixed with acid citrate dextrose (citric acid/sodium citrate/dextrose, 3:6:4; w/w/w) in 5:1 volumetric ratio (blood/anticoagulant), followed by dextran sedimentation and hypotonic lysis to remove red blood cells. Then, the cell pellet was suspended in 2 ml of PBS and subjected to density gradient centrifugation using Histopaque-1077 for 30 min, 210*g* at 4 °C, after which the neutrophils settled at the bottom as a cell pellet. This pellet was washed twice with PBS for 6 min, centrifuged at 210*g*, and re-suspended in HBSS buffer without cations containing 2% HSA or DMEM. The cells were counted using a Neubauer chamber and the required cell density was adjusted using HBSS/DMEM. Wright and Giemsa staining was used to determine the purity of the cells, which was >95%.

### Quantification of NETosis by Hoechst stain and MPO-DNA ELISA

NETosis was quantified by Hoechst stain^32^. The neutrophils (2 × 10^5^ cells per ml) were seeded on 13 mm round coverslips placed in 24-well culture plates in 500 μl of DMEM with 2% HSA and allowed to adhere to the coverslips for 30 min at 37 °C and 5% CO_2_. Then, the cells were independently stimulated with *E. carinatus* venom (5–50 μg ml^−1^) for 180 min to assess the dose-dependent response. To assess the time-dependent response, the *E. carinatus* venom (25 μg ml^−1^) was incubated for different time intervals from 0 to 180 min. PMA (50 nM) served as a positive control. The cells were then fixed with 4% paraformaldehyde, followed by Hoechst 33342 staining (1:10,000). For NET quantification, images were acquired on a BA410 fluorescence microscope (Motic) attached to a DS-Qi2 monochrome CMOS sensor camera (Nikon) using a CCIS EC-H Plan achromatic × 40/0.65 objective lens and NIS-Elements D software (Version 4.3.00). The NET percentage was determined in 12 non-overlapping fields per coverslip. The images were analysed using ImageJ software. The average NET percentage was calculated from triplicate experiments. The experimenter was blinded to the treatment conditions during the analysis.

To quantify the NETs in the cell supernatant, we used a capture ELISA (Cell Death ELISA^PLUS^, Roche) method based on capture of the MPO-associated DNA^53^. NETosis was induced using either *E. carinatus* or *N. naja* venom (5–50 μg ml^−1^), as described above. PMA (50 nM) served as a positive control. Then, the reaction mixture was centrifuged at 20*g* for 5 min at room temperature and the supernatant was separated. The anti-MPO mAb (1:200, 50 μl) was coated onto 96-well plates overnight at 4 °C. After three washes (300 μl each), 20 μl of the reaction supernatant and 80 μl of incubation buffer containing peroxidase-labelled anti-DNA mAb (1:25) were added to the wells and incubated by shaking at 300 r.p.m. for 2 h at room temperature. Then, the wells were washed three times (with PBS, 300 μl each) and 100 μl of ABTS was added. After 20 min of incubation at room temperature in the dark, the absorbance was measured at 405 nm. To calculate NET percentage, fluorescence obtained from cells lysed with 0.5% Triton X-100 was considered as 100% NET formation. To demonstrate venom-induced NOX-dependent and/or NOX-independent NETosis, neutrophils were independently pre-incubated with DPI (20 μM) and/or DNP (750 μM) for 60 min at 37 °C and then incubated with *E. carinatus* venom (50 μg) for 180 min at 37 °C. PMA (50 nM) and A23187 (5 μM) were used as a positive control for inhibition of the NOX-dependent and NOX-independent pathways, respectively.

### Detection of ROS

*E. carinatus* venom-stimulated ROS in neutrophils was quantified using DCFDA^29^. The neutrophils (2 × 10^5^ cells per ml) were incubated with increasing doses of *E. carinatus* venom (5–50 μg ml^−1^) for 30 min at 37 °C. After incubation, DCFDA (10 μM) was added to determine ROS. The fluorescence was measured at 530 nm after exciting at 480 nm by using Varioskan multimode plate reader (Thermo Scientific) and expressed as pmol DCF formed per mg protein. Further to demonstrate venom-induced NOX-dependent and/or NOX-independent ROS production, neutrophils were independently pre-incubated with DPI (20 μM) and/or DNP (750 μM) for 60 min at 37 °C and then stimulated with *E. carinatus* venom (50 μg) for 30 min at 37 °C. PMA (50 nM) and A23187 (5 μM) were used as a positive control.

### Immunocytochemistry

Neutrophils (2 × 10^5^ cells per ml) were seeded on 13 mm round coverslips placed in 24-well culture plates in 500 μl of DMEM with 2% HSA and allowed to adhere to the coverslips for 30 min at 37 °C and 5% CO_2_. Then, the cells were independently stimulated with PMA (50 nM) and the *E. carinatus* venom (25 and 50 μg ml^−1^) for 2.5 h, fixed using 4% paraformaldehyde, permeabilized using 1% Triton X-100 and blocked with 1% bovine serum albumin for 1 h at room temperature. The cells were incubated with a primary antibody against H3Cit (1:1,000) overnight at 4 °C and then with AlexaFluor 488-conjugated goat anti-rabbit IgG (1:1,500) for 2 h at room temperature. Hoechst 33342 (1:10,000) was used to stain for DNA. The images were acquired on a BA410 fluorescence microscope (Motic) attached to a DS-Qi2 monochrome CMOS sensor camera (Nikon) using a CCIS EC-H Plan Achromatic × 20 or × 40/0.65 objective lens and NIS-Elements D software (Version 4.3.00 64-bit). Images were analysed using the ImageJ software.

### Scanning electron microscopy

Neutrophils (2 × 10^5^ cells per ml in 500 μl DMEM with 2% HSA) were seeded on 13 mm round coverslips, placed in 24-well culture plates and allowed to attach to the coverslips for 30 min at 37 °C. The neutrophils were then stimulated with *E. carinatus* venom (50 μg ml^−1^) for 2.5 h at 37 °C, fixed in 2.5% glutaraldehyde and post-fixed in 0.5% osmium tetroxide for 30 min. Then, the coverslips were incubated in 1% tannic acid for 30 min, with 0.5% osmium tetroxide for 30 min and dehydrated with a graded series of alcohol (30–100%) for 5 min each. The coverslips were dried in a desiccator for 24 h and the coverslips containing specimens were coated with a carbon layer using a thin layer evaporator. The samples were visualized using a Zeiss EVO LS15 scanning electron microscope.

### Venom-induced mouse tail tissue destruction and lethality

*E. carinatus* or *N. naja* venom (LD_50_/LD dissolved in 50 μl PBS) was subcutaneously administered to the groups of mice (*n*=10) 3 cm distal to the base of the tail. The lethal dose of venom was determined in a pilot study (*E. carinatus* venom LD_50_=1 mg per kg body weight and LD=1.5 mg per kg body weight and *N. naja* venom LD=0.75 mg per kg body weight). The time of death and tail injuries were recorded for each mouse. The severity of the tail injury was judged visually and scored according to a 10 point scale; 0=no injury, 1=oedema, 2=oedema with minor haemorrhage, 4=oedema with haemorrhage causing less than 25% tail discolouration, 6=oedema and major haemorrhage or wound causing 25–50% tail discolouration, 8=oedema and major haemorrhage or wound causing 50–75% tail discolouration, 10=oedema and major haemorrhage or wound causing more than 75% tail discolouration. The tail injury observations were recorded every day for 15 days after venom injection. To assess the effects of DNase 1 on tail haemorrhage, *E. carinatus* venom (LD_50_/LD) was co-injected independently with 25, 50 or 100 U DNase 1. In the challenge study, 100 U DNase 1 was administered ∼5 mm distal to the venom injection site 30, 60, 90, 120 or 180 min after the *E. carinatus* venom (LD_50_) injection. To assess the effect of DNA on venom lethality, *E. carinatus* venom (LD_50_/LD) was incubated with calf thymus DNA in a 1:1 (w/w) ratio for 10 min at 37 °C and injected subcutaneously into the tail. Appropriate controls were maintained according to the assay requirements.

### Histopathological studies

The venom-induced tail tissue destruction and the presence of NETs in the venom-injected tail tissues were examined in H&E-stained tissue sections. The respective tissues were dissected from the venom injection site, fixed overnight in buffered formalin and subjected to dehydration with different grades of alcohol and chloroform mixture. The processed tissues were embedded in molten paraffin wax, and 10-μm-thick sections were prepared using a microtome. The sections were stained with H&E, observed under an Axio Imager.A2 microscope (Zeiss) and photographed.

### Immunohistochemistry

The localization of H3Cit, Ly6G/lactoferrin and DNA in mouse tail tissue was examined by immunofluorescence microscopy. The mouse tail tissue was dissected from the *E. carinatus* venom-injected normal and neutropenic mice, processed, embedded in solidifying paraffin wax and cut into 10-μm-thick cross/longitudinal sections. The sections were deparaffinized by incubating the slides overnight at 55 °C and subjected to xylene clearance for 5 min. Furthermore, the sections were rehydrated with different grades of alcohol (100–50%). The antigens were retrieved by incubating the slides with Tris-EDTA buffer (pH 9.0) in a steamer for 45 min. The tissues were permeabilized using 1% Triton X-100 and incubated with primary antibodies against H3Cit (1:1,000) overnight at 4 °C, followed by incubation with an AlexaFluor 488-conjugated goat anti-rabbit IgG antibody (1:500) for 2 h in the dark at room temperature. The sections were then incubated with an AlexaFluor 647-conjugated anti-mouse Ly6G antibody (1:200) for 3 h at room temperature or anti-mouse lactoferrin antibody (1:500) for overnight at 4 °C, followed by incubation with an AlexaFluor 647-conjugated goat anti-mouse IgG antibody (1:500) for 2 h in the dark at room temperature. Hoechst 33342 (1:10,000, 1 μg ml^−1^) was used to stain DNA. Images were acquired with a BA410 fluorescence microscope (Motic) attached to a DS-Qi2 monochrome CMOS sensor camera (Nikon) using a CCIS EC-H plan achromatic × 10/0.25 and × 40/0.65 objective lens and NIS-Elements D software (Version 4.3.00), or using a confocal microscope (Zeiss LSM 510 Meta). The appropriate controls were maintained.

The accumulation of venom toxins in *E. carinatus*/*N. naja* venom-injected tail tissues after 8 h of venom injection in the presence and absence of DNase 1/actin were detected using the appropriate rabbit polyclonal antibodies (2 μg ml^−1^), followed by an AlexaFluor 488-conjugated goat anti-rabbit IgG secondary antibody (1:500). The appropriate controls were maintained.

### Rabbit immunization and IgG purification

Rabbits were independently immunized against *E. carinatus* and *N. naja* venoms, and IgGs were purified^54^. *E. carinatus* (200 μg) or *N. naja* (100 μg) venoms were diluted in 100 μl PBS, thoroughly mixed with an equal volume of Freund's complete adjuvant and intradermally injected into female rabbits at several sites. Three booster doses of venom were administered at the same concentration and an equal volume of Freund's incomplete adjuvant at weekly intervals. Blood was drawn from the marginal ear vein on the ninth day after the third booster dose and allowed to coagulate for 24 h at 8–10 °C to obtain the antiserum. The antiserum was subjected to ammonium sulfate precipitation to obtain the crude IgG fraction, which was then subjected to Protein A-agarose column chromatography. The column was equilibrated with PBS and 5 mg of the crude IgG fraction in 2 ml of PBS was loaded and eluted using 0.2 M glycine-HCl buffer, pH 2.9. After reading the optical density at 280 nm, 1 ml aliquots were collected, pooled and then neutralized using 1 M Tris-HCl buffer, pH 8.0. The samples were subjected to dialysis against PBS. Aliquots of antibodies with 2 mg ml^−1^ concentration were prepared and stored at −20 °C and used for the study.

### Neutropenic mouse model

Neutropenia was induced in mice using cyclophosphamide^55^. Briefly, female Swiss albino mice were intraperitoneally injected with cyclophosphamide in two doses totaling 250 mg kg^−1^. Initially, 150 mg kg^−1^ was administered in 500 μl saline as the first dose on day 1, and the second dose of 100 mg kg^−1^ was administered on day 4. Blood samples were drawn from the retro-orbital plexus on days 4 and 5, and subjected to total and differential cell counts using a Neubauer chamber and microscopic examination of Wright-stained smears. Then, the neutropenic mice were used on day 4, where complete neutrophil depletion was found (Supplementary Table 1), to determine the tail tissue destruction activity and lethality (*n*=10) of the *E. carinatus* venom (LD_50_) in the presence and absence of DNase 1 (100 U) as described above.

### Western blot analysis

In the *ex vivo* experiment, the levels of H3Cit, histone H3 and PAD4 in human neutrophils incubated with 5, 25 and 50 μg ml^−1^ of *E. carinatus* venom for 2.5 h at 37 °C were observed by western blotting. In the *in vivo* studies, the levels of H3Cit/histone H3/MPO were determined in mice tail homogenates. The mice were divided into three different treatment groups: (i) *E. carinatus* venom (LD_50_) was injected into the mice at different time intervals (2, 4, 8, 16 and 12 h; 3 and 10 days); (ii) *E. carinatus* venom (LD_50_) was injected into normal and neutropenic mice for 8 h and (iii) *E. carinatus* venom (LD_50_) was injected into mice in the presence or absence of DNase 1 (100 U) for 8 h. The accumulation of venom in tail tissue was observed using western blots of tail tissue homogenates from mice injected with *E. carinatus* venom (LD_50_) in the presence or absence of DNase 1 (100 U) and mice injected with *N. naja* venom in the presence or absence of actin (50 μM) 8 h after injection using rabbit raised polyclonal antibodies raised against respective snake. Briefly, the treated human neutrophils and mouse tail portions (3 cm length of tail, with the injection site at the centre, were collected after the mice were anaesthetized and killed at the indicated time intervals in the respective experiments) were snap frozen and homogenized in RIPA buffer with protease inhibitor cocktails on ice. The homogenates were centrifuged at 15,000*g* for 15 min at 4 °C and equal amounts of protein were fractionated on SDS–PAGE and electroblotted onto polyvinylidene difluoride (PVDF) membranes. The blots were then incubated with primary antibodies (anti-H3Cit (1:750), anti-H3 (1:750), anti-PAD4 (1:2,000), anti-MPO (1:750), anti-*E. carinatus* (2 μg ml^−1^) and anti-*N. naja* (2 μg ml^−1^)) overnight at 4 °C and subsequently with the appropriate horseradish peroxidase-conjugated secondary antibody (1:5,000) for 2 h at room temperature. The blots were developed with an enhanced chemiluminescence substrate and visualized (Alliance 2.7, Uvitec). To confirm equal loading, the glyceraldehyde 3-phosphate dehydrogenase (GAPDH) levels were observed by incubating the membrane with an anti-GAPDH antibody (1:1,000). For the venom accumulation study, the electroblotted membranes were stained with Ponceau to confirm equal protein loading. ImageJ software was used to quantify the blots. Images have been cropped for presentation. Uncropped images are presented in Supplementary Fig. 11.

### Detection of DNase activity of the venom

The DNase activity of the venom was determined by agarose gel electrophoresis/DNase radial diffusion assay. Briefly, 750 ng of calf thymus DNA was independently incubated with the *N. naja* (5–50 μg ml^−1^) venom for 60 min, at 37 °C in a final volume of 50 μl PBS. The reaction mixture was subjected to electrophoresis on 0.8% agarose gels at 50 V in TAE buffer (40 mM Tris-base and 1 mM EDTA, pH 8.0) for 1 h. Calf thymus DNA that had been treated with DNase 1 (5 U) served as a positive control. After electrophoresis, the gel was visualized and photographed on a ultraviolet transilluminator (Alliance 2.7, Uvitec). The *N. naja* venom DNase activity was inhibited by incubating the sample with actin (5–25 μM) for 10 min at 37 °C. Image has been cropped for presentation. Uncropped image is presented in Supplementary Fig. 11.

For radial diffusion assay, molten agarose (5 ml, 2.1%) containing herring sperm DNA (1 mg ml^−1^) and ethidium bromide (1 μg ml^−1^) was poured into wells of six-well culture plates and allowed to solidify. Seven-millimetre diameter wells were created at the centre of the dishes and loaded with *E. carinatus* venom (50–500 μg per well) for 24 h at 37 °C. DNase 1 (10 U) served as the positive control. After incubation, the plates were visualized and photographed on a ultraviolet transilluminator (Alliance 2.7, Uvitec).

### Effect of *E. carinatus* venom on serum DNase activity

Serum DNase activity was determined using the radial diffusion method. Molten agarose (5 ml, 2.1%) containing herring sperm DNA (1 mg ml^−1^) and ethidium bromide (1 μg ml^−1^) was poured into 35 mm diameter Petri dishes and allowed to solidify. Seven-millimetre diameter wells were created at the centre of the dishes and loaded with human serum (200 μl), which was independently pre-incubated with 250, 500 and 1,000 μg of *E. carinatus* venom for 1 h at 37 °C. DNase 1 (10 U) served as the positive control. After 24 h of incubation at 37 °C, the plates were visualized and photographed on a ultraviolet transilluminator (Alliance 2.7, Uvitec).

### DNA-venom capture ELISA

The interaction between DNA and *E. carinatus* venom was studied using DNA–venom capture ELISA with Cell Death ELISA^PLUS^, Roche. Neutrophils (1 × 10^5^ cells per 500 μl) were incubated with *E. carinatus* venom (25, 50 and 100 μg) for 2.5 h to ensure NET formation and binding of the *E. carinatus* venom to NET DNA. Next, the DNA–histone complex provided in the assay kit (40 μl) was incubated with *E. carinatus* venom (50 μg) in a final volume of 500 μl made up with incubation buffer for 2.5 h to serve as a positive control. The concentrations of the DNA–histone complex and *E. carinatus* were determined in our pilot study, where 40 μl of the DNA–histone complex bound to a maximum of 50 μg of *E. carinatus* venom. After incubation, the samples were centrifuged at 20*g* for 5 min at room temperature, and 20 μl of the supernatant was added to streptavidin-coated 96-well plates, along with 80 μl of an anti-histone-biotin antibody (1:20) for 2 h, followed by shaking at 300 r.p.m. at room temperature. The plate was washed (three times with incubation buffer, 300 μl each) and incubated with a rabbit polyclonal antibody against *E. carinatu*s venom (1:1,000) for 2 h, followed by shaking at 300 r.p.m. at room temperature. Then, the plate was washed (three times with incubation buffer, 300 μl each) and incubated with an horseradish peroxidase-conjugated anti-rabbit secondary antibody (1:5,000) for 2 h by shaking at 300 r.p.m. at room temperature in the dark. The wells were again washed (three times with incubation buffer, 300 μl each) and 100 μl of the ABTS peroxidase substrate was added. After 20 min of incubation at room temperature in the dark, the absorbance was measured at 405 nm using Varioskan multimode plate reader (Thermo Scientific). The percent increase in the absorbance with respect to the control indicated the binding of *E. carinatus* venom toxins to the DNA–histone complex or NETs.

### Detection of DNA–venom interaction by non-denaturing PAGE

The interaction between DNA and *E. carinatus* venom was also studied by electrophoresis. We used the amplified PCR product (160 bp) that was produced by multiplex PCR using the mecA P4 (**5′-**TCCAGATTACAACTTCACCAGG-**3′**) and mecA P7 (**5′-**CCACTTCATATCTTGTAACG-**3′**) primers as previously described^56^. The reaction was carried out with a DreamTaq Green DNA Polymerase kit consisting of 200 μM dNTPs, 1.25 U DreamTaq DNA polymerase and 100 ng template DNA (Genomic source *S. aureus*). PCR amplification was performed in a Surecycler 8800 (Agilent Technologies) using the following conditions: initial denaturation for 15 min at 95 °C; 35 cycles consisting of denaturation for 30 s at 95 °C, annealing for 30 s at 53 °C, elongation for 1 min at 72 °C and post-extension for 5 min at 72 °C. The amplified fragment/amplicon (250 ng) was incubated with increasing concentrations of *E. carinatus* venom (5–50 μg) for 1 h at 37 °C in a final reaction volume of 20 μl. Then, the reaction mixture was separated on non-denaturing PAGE (7.5%) using 0.5 × Tris-borate-EDTA buffer, pH 8.5, with a constant voltage of 50 V for 4 h. The gel was visualized on a ultraviolet transilluminator (Alliance 2.7 Uvitec) after staining with ethidium bromide (1 μg ml^−1^) for 30 min at room temperature.

The interaction between the DNA and the *E. carinatus* venom was disrupted by the addition of an equal amount of chloroform and isoamyl alcohol (24:1, v/v) followed by two volumes of pre-chilled isopropanol and incubation overnight at −20 °C. The samples were then centrifuged at 18,000*g* for 10 min and the pellets were dissolved in sterile distilled water and resolved on a non-denaturing PAGE gel as described above. Images have been cropped for presentation. Uncropped images are presented in Supplementary Fig. 11.

### Protein concentration measurement

The protein concentrations were determined using the method described by Lowry *et al*.^57^.

### Statistics

The data are presented as the mean±s.e.m. of at least three independent experiments and were analysed using a two-tailed Student's *t*-test (unpaired), one-way analysis of variance, followed by Dunnett's *post-hoc* test or Bonferroni *post hoc* test for multiple comparisons as applicable. Lethality was analysed using the log-rank test after constructing Kaplan–Meier curves. All analyses were done using GraphPad Prism software (Version 5.0). The results were considered significant when *P*<0.05.

## Additional information

**How to cite this article:** Katkar, G. D. *et al*. NETosis and lack of DNase activity are key factors in *Echis carinatus* venom-induced tissue destruction. *Nat. Commun.* 7:11361 doi: 10.1038/ncomms11361 (2016).

## Supplementary Material

Supplementary Information Supplementary Figures 1-11 and Supplementary Table 1
