# Supplementary material for: NETosis and lack of DNase activity are key factors in Echis carinatus venom-induced tissue destruction
Source: Nat Commun. 2016 Apr 19;7:11361. doi: 10.1038/ncomms11361 (PMC4838891; doi:10.1038/ncomms11361)
Supplement: Supplementary Information — Supplementary Figures 1-11 and Supplementary Table 1 [file ncomms11361-s1.pdf]

Supplementary information

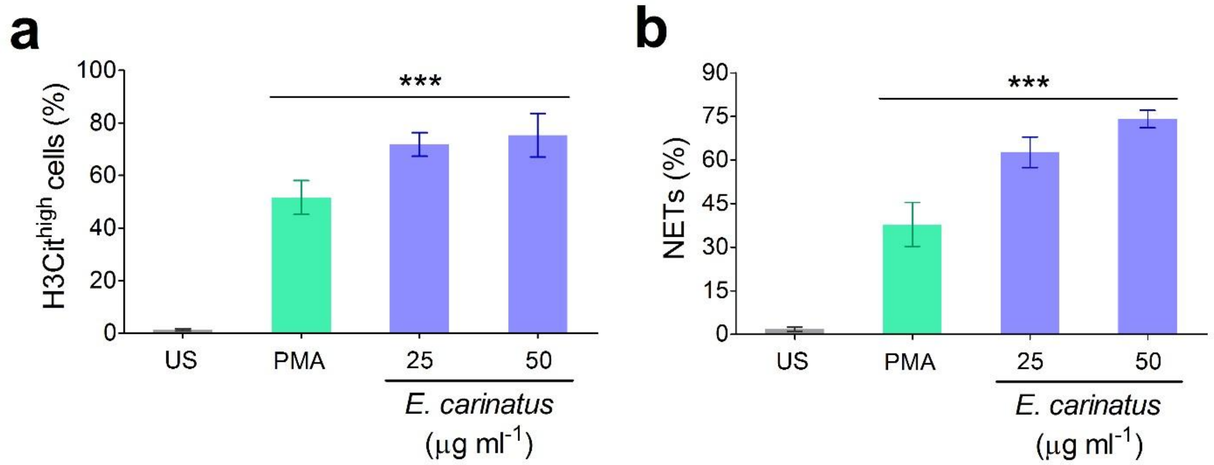

Supplementary Figure 1: ***E. carinatus* venom induces NETosis.** Neutrophils (2×10^5 cells ml^{-1}) were seeded on 13 mm round cover slips placed in 24-well culture plates in 500 µl of DMEM with 2% HSA and allowed to adhere to the cover slips for 30 min at 37 °C and 5% CO2. Cells were independently stimulated with PMA (50 nM) and the *E. carinatus* venom (25 and 50 µg ml^{-1}) for 2.5 h. The cells were incubated with a primary antibody against H3Cit (1:1,000) overnight at 4 °C and then with Alexa Fluor®488-conjugated goat anti-rabbit IgG (1:1,500) for 2 h at room temperature. Hoechst 33342 (1:10,000) was used to stain for DNA. The images were acquired on a BA410 fluorescence microscope (Motic, Japan) attached to a DS-Qi2 monochrome CMOS sensor camera (Nikon, Japan) using a CCIS EC-H Plan Achromatic 20× or 40×/0.65 objective lens and NIS-Elements D software (Version 4.3.00 64-bit). Images were analyzed using the ImageJ software. (a) Percentage of cell that were hypercitrunillated at histone H3 ($H3Cit^{high}$) and (b) produced NETs when exposed to *E. carinatus* venom (25 and 50 µg ml^{-1}) and PMA (50 nM) for 3 h. H3Cit, citrullinated histone 3. US, unstimulated cells. Data are mean ± S.E.M. (n = 4). ***$p < 0.001$ versus US. One way ANOVA followed by Dunnett's post-hoc test. PMA served as positive control.

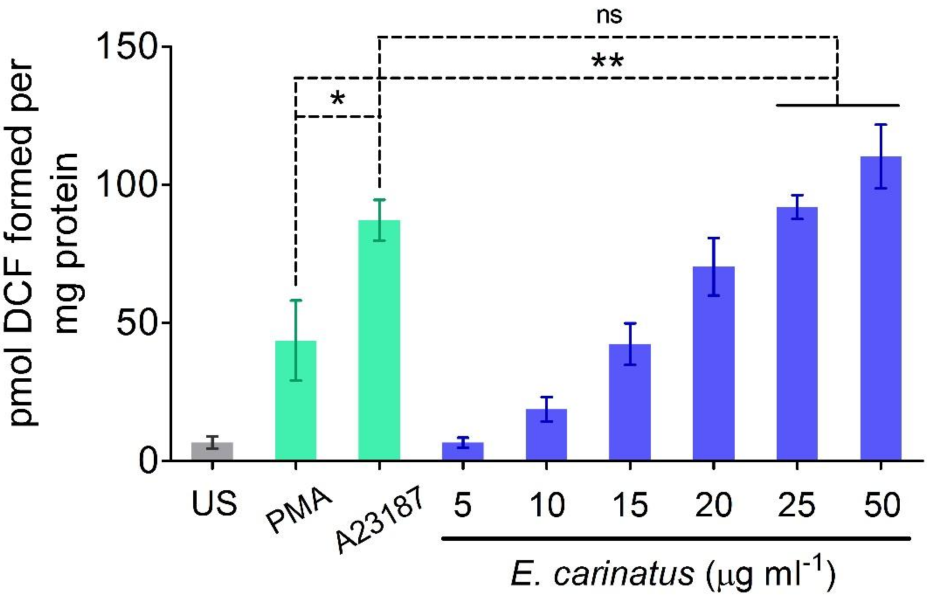

Supplementary Figure 2: *E. carinatus* venom mediated ROS production. Graph represents the level of ROS in neutrophils incubated with *E. carinatus* venom (5-50 μg ml^{-1}). The data are presented as mean ± S.E.M. (n = 4). *$p < 0.05$, **$p < 0.01$; ns, non-significant. One-way ANOVA, followed by Bonferroni post-hoc test. PMA (50 nM) and A23187 (5 μM) were served as positive control.

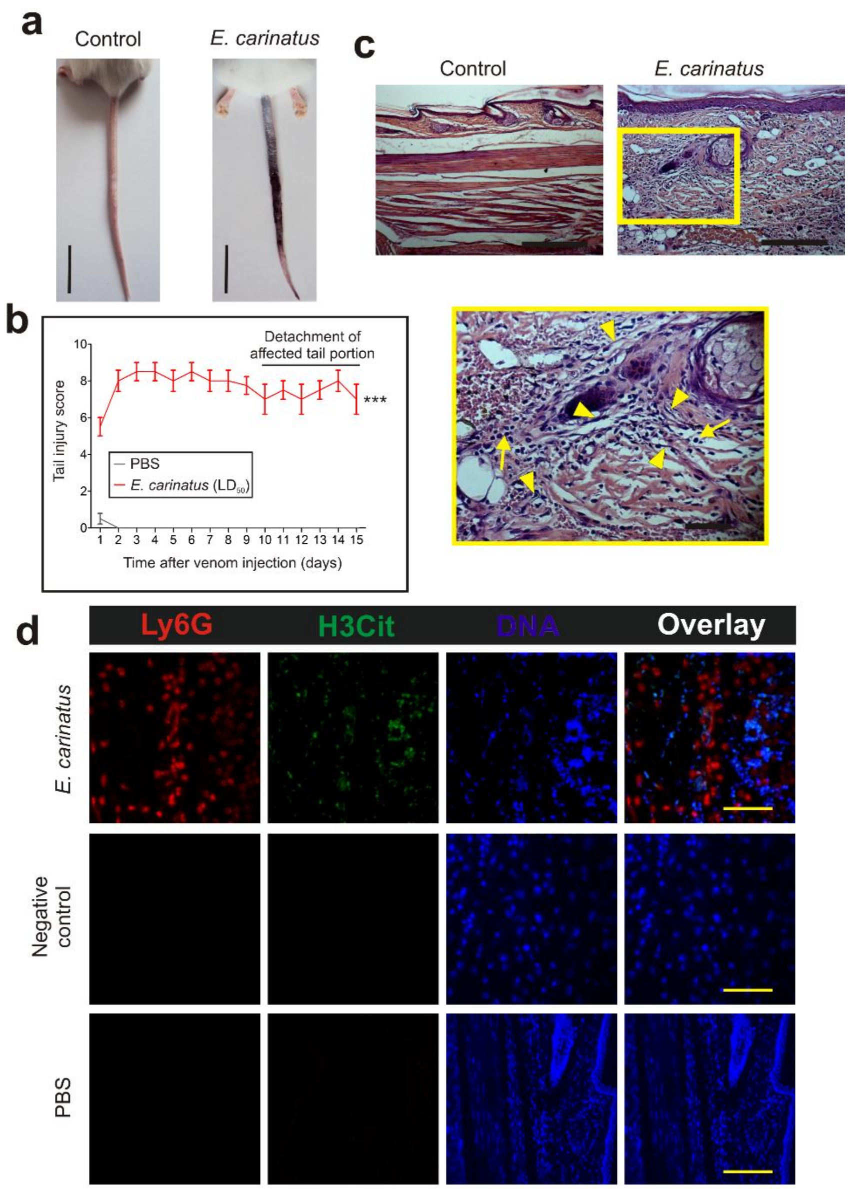

Supplementary Figure 3: *E. carinatus* venom induces local tissue destruction in mice tail. (a) Representative photographs of *E. carinatus* venom (LD_{50}) injected mouse tail showing wounded tail (right) as compared to PBS injected control tail (left) after 8 h of venom injection. Scale bars, 2 cm. (b) The corresponding tail injury score. Data are mean ± S.E.M. n = 6 for PBS injected control mice; n = 10 for venom injected mice. ***$p < 0.001$ versus PBS injected control. Student's t-test. (c) Photomicrographs of H & E stained PBS injected control mouse tail tissue section (top, left) showing no neutrophils and no detectable NETs, the *E. carinatus* venom

(LD_{50}) injected tail tissue section (top, right). Area enclosed by the yellow box is magnified and shown at the bottom showing large accumulation of neutrophils (Yellow arrows) and NETs (Yellow arrowheads). Scale bars, 100 µm (Top), 50 µm (enlarged image, bottom). (d) Neutrophil H3Cit is observed only in *E. carinatus* venom-injected mice tail tissue (top row). No neutrophils or H3Cit were detected in the PBS-injected mice tail tissue (middle row). Ly6G and H3Cit signals were negative in the control without primary antibodies (reference to Figure 3b).

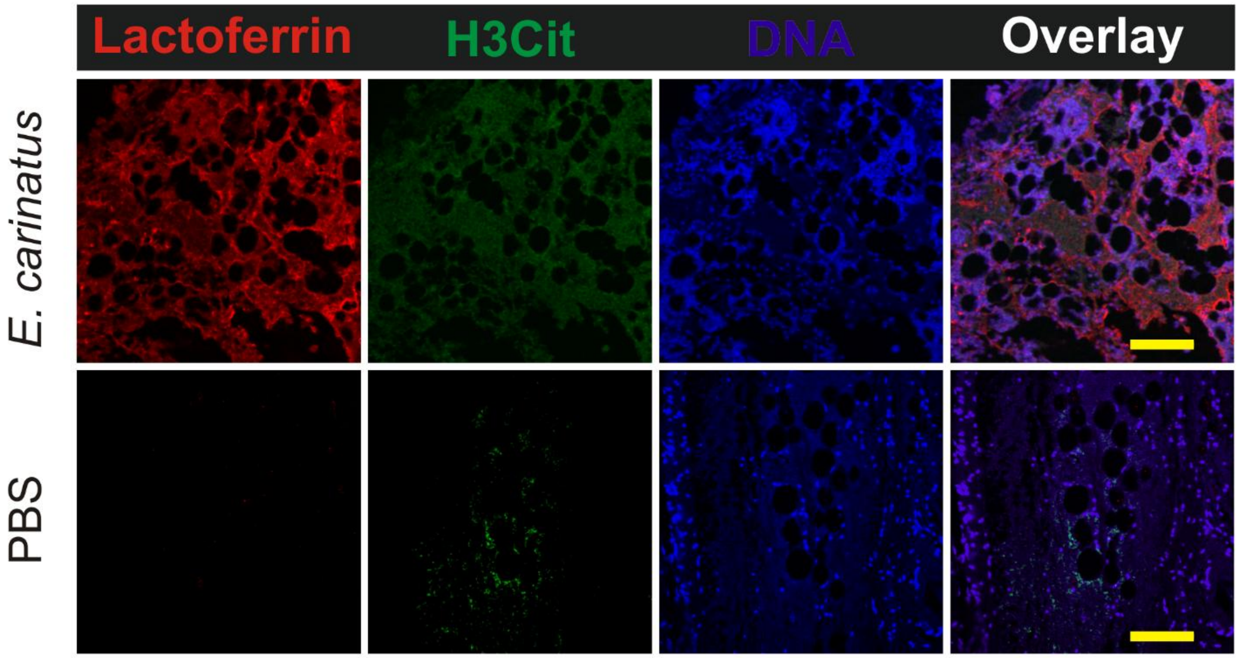

Supplementary Figure 4: *E. carinatus* venom induces NETs in mice tail tissue. Representative confocal images showing presence of lactoferrin and H3Cit in *E. carinatus* venom (LD_{50})-injected mice tail tissue (top row). The lactoferrin, H3Cit and DNA co-localization was not detected in the PBS-injected mice tail tissue (bottom row). However, H3Cit showed some non-specific binding in mice tail tissue (reference to Figure 3c). Scale bars, 100 µm.

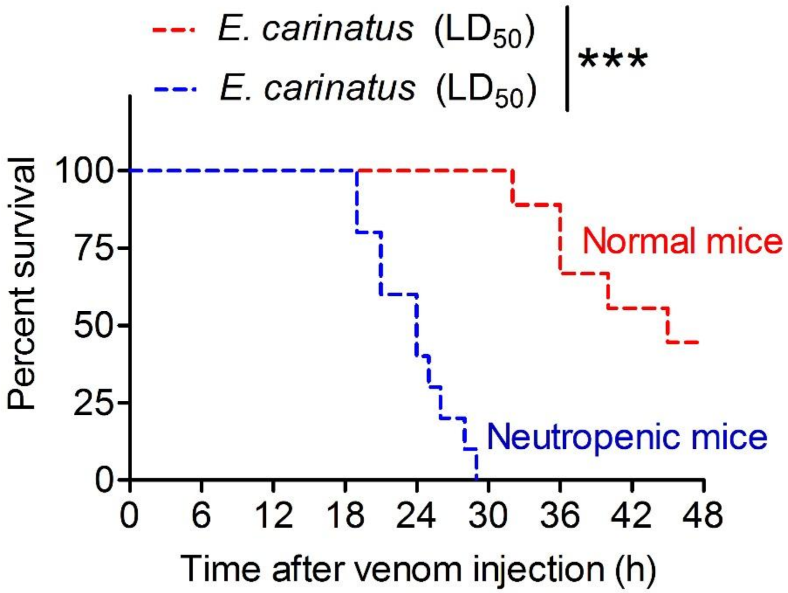

Supplementary Figure 5: Kaplan-Meier survival curves showing differential lethal effect of *E. carinatus* venom. *E. carinatus* venom (LD_{50}) injected to normal mice (red line) and neutropenic mice (blue line). (n = 10). ***$p < 0.001$. Log-rank test.

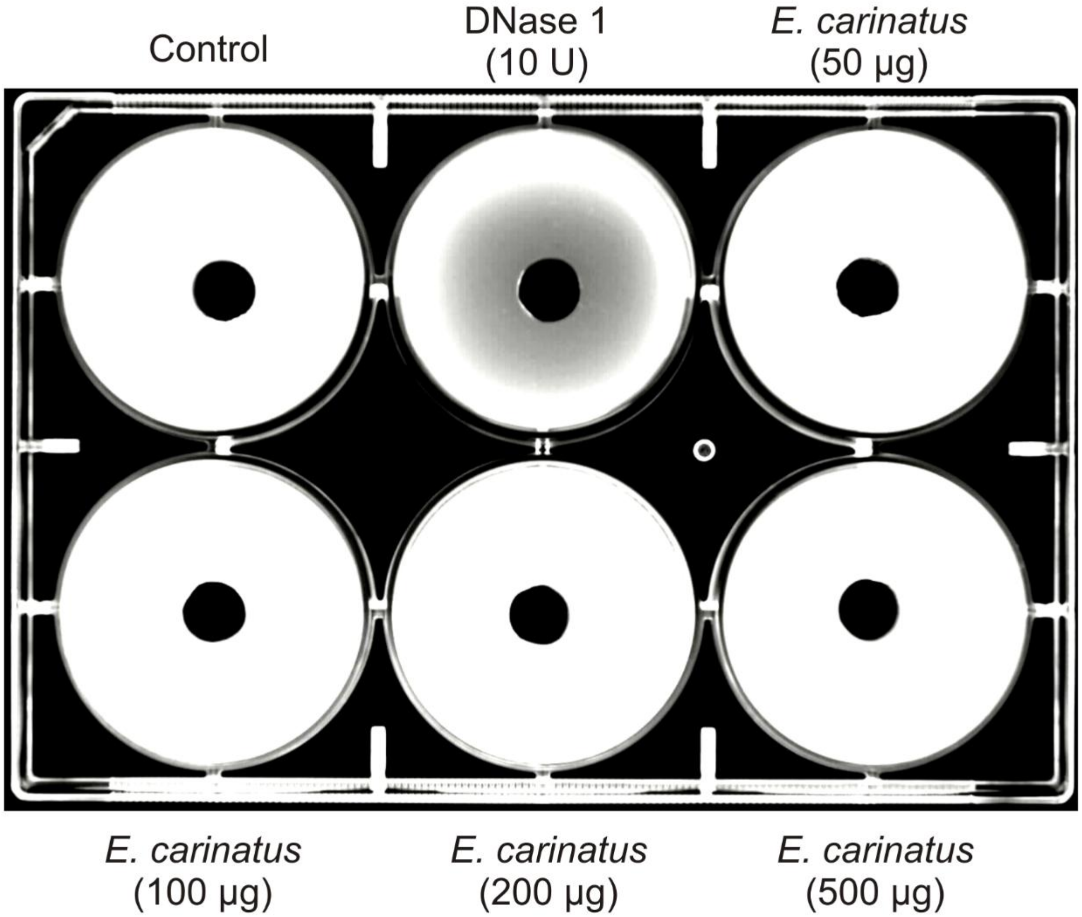

Supplementary Figure 6: DNase radial diffusion assay showing lack of activity zone in presence of increasing doses of *E. carinatus* venom (50-500 µg per well), which was similar as that of PBS control. DNase 1 (10 U) is used as positive control.

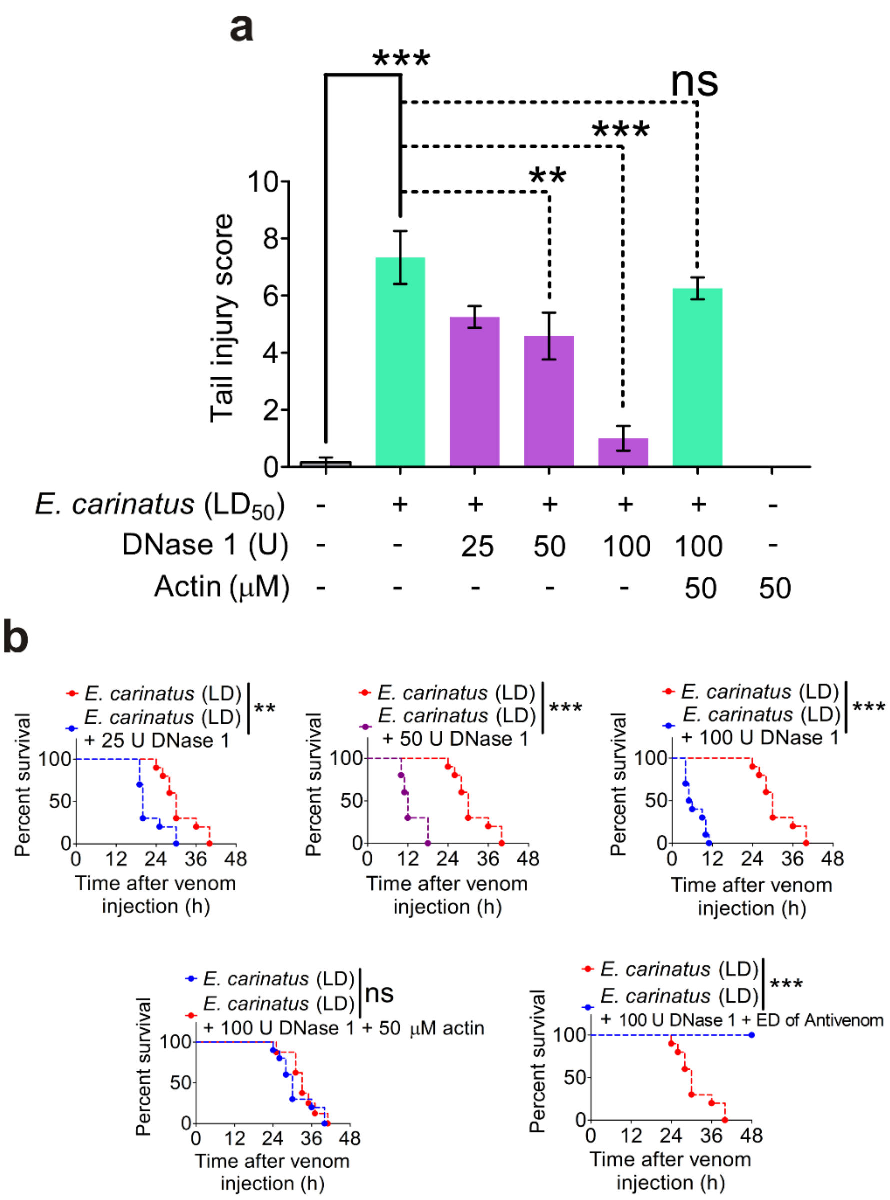

Supplementary Figure 7: Co-injection of *E. carinatus* venom and DNase 1 abrogated local tissue damage and increases lethal potency. (a) Tail injury score of *E. carinatus* venom (LD_{50}) alone, in presence of DNase 1 (25-100 U) and in presence of actin (50 µM) pre-treated DNase 1 (100 U). PBS and actin (50 µM) served as controls. Data are mean ± S.E.M. (n = 4). $^{**}p < 0.01$, $^{***}p < 0.001$ versus PBS injected control. ns, non-significant. One way ANOVA followed by Dunnett's post-hoc test. (b) All the experiment in this group were performed simultaneously but

divided into five graphs (top three and bottom two) for better comparison and understanding. Kaplan-Meier survival curves, *E. carinatus* venom, LD (red line) in all the graphs, co-injection of *E. carinatus* venom (LD) with 25 U DNase 1 (blue line), 50 U DNase 1 (green line), 100 U DNase 1 (violet line), 100 U DNase 1 pre-incubated with 50 µM actin (gray line), and 100 U DNase 1 followed by ED AV (black line). ED AV, effective dose of antivenom. (n = 10). $^{**}p < 0.01$, $^{***}p < 0.001$ and ns, non-significant between groups. Log-rank test.

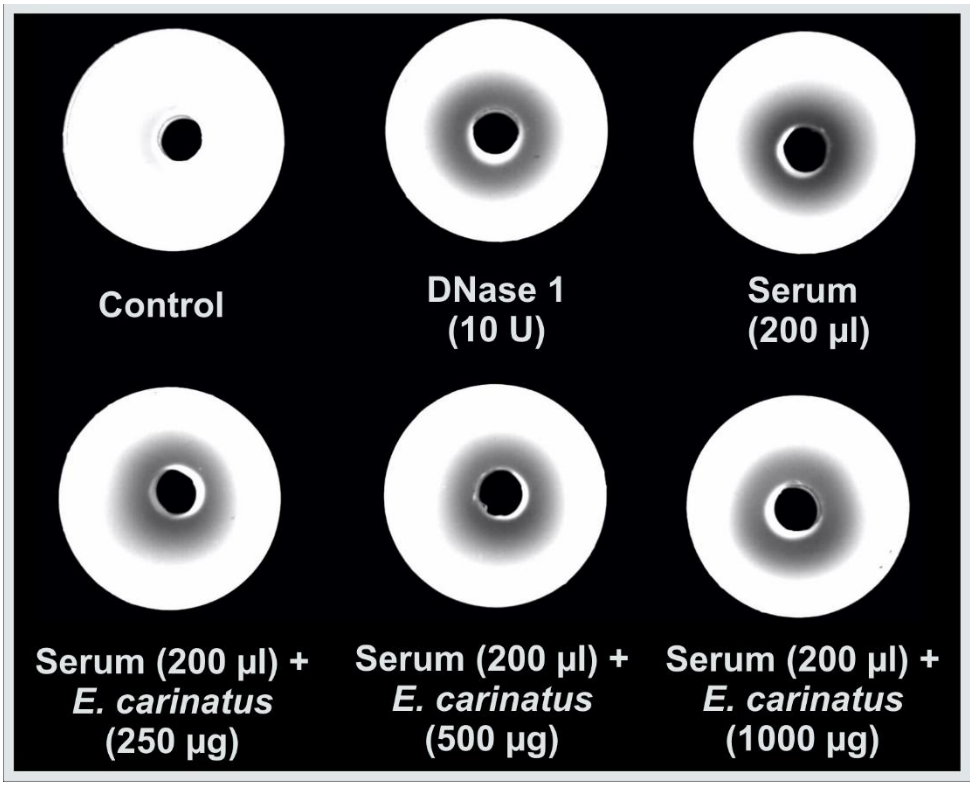

Supplementary Figure 8: **Effect of *E. carinatus* venom on serum DNase activity in a DNase radial diffusion assay.** Serum DNase activity was observed in presence and absence of *E. carinatus* venom. Serum (200 µl) was pre-incubated with increasing dose of *E. carinatus* venom (250, 500 and 1000 µg per well) in final volume of 250 µl for 1 h at 37 °C and the images were acquired on a UV transilluminator after 24 h of incubation at 37 °C. DNase 1 (10 U) is used as positive control.

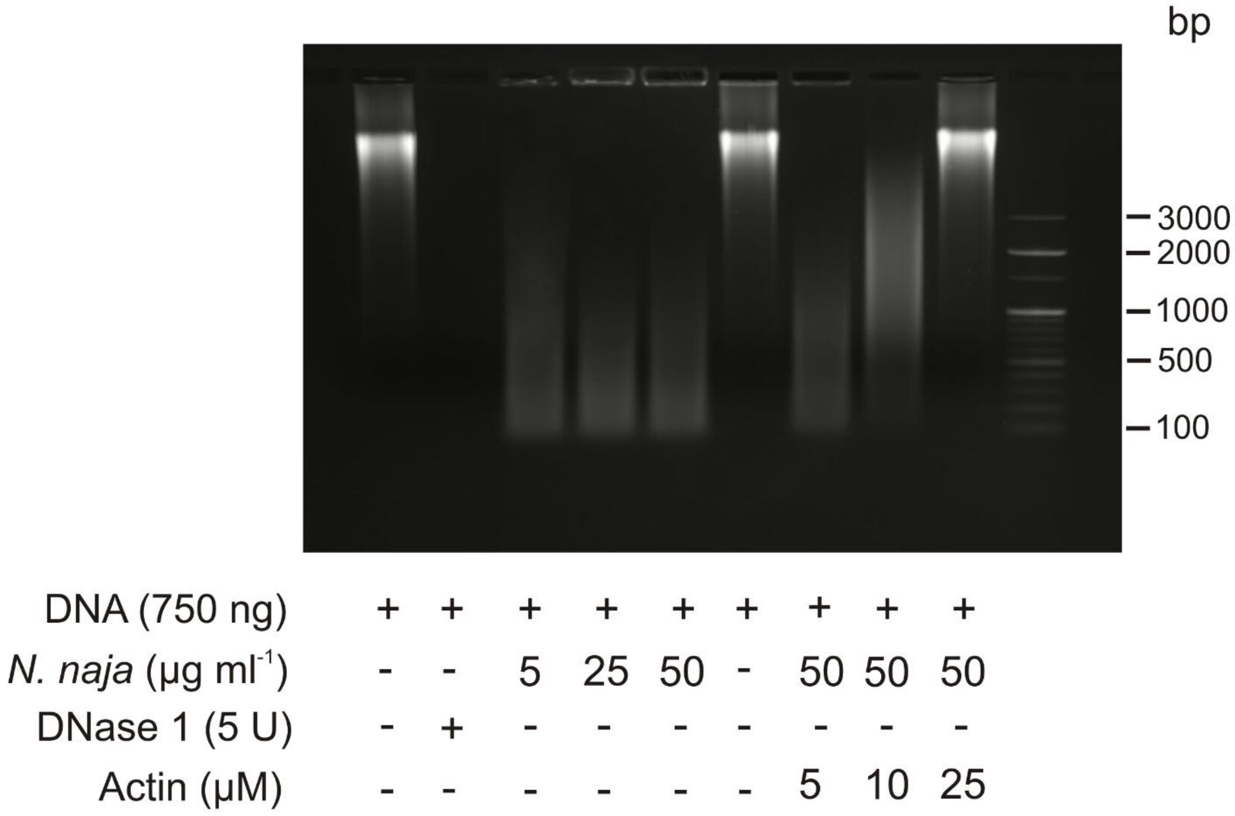

Supplementary Figure 9: DNase activity of *N. naja* venom. Agarose gel electrophoresis showing cleaved DNA fragments in presence of increasing doses of *N. naja* venom (5-50 $\mu g\ ml^{-1}$), and restoration of DNA band when venom pre-incubated with increasing concentration of actin which was similar as that of untreated DNA control. DNase 1 (5 U) served as positive control.

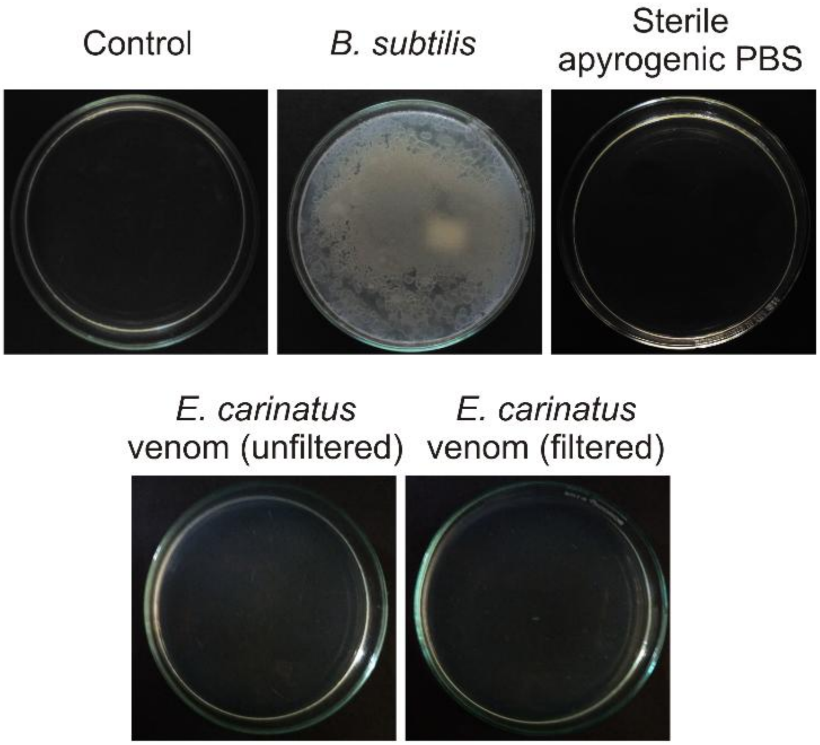

Supplementary Figure 10: Bacterial agar culture plates show *E. carinatus* venom (200 µg) lacks bacterial contamination. Unfiltered venom (bottom, left) and filtered venom (bottom, right) showing no bacterial growth on agar culture plates after incubation of 24 h. *B. subtilis* was served as positive control (top, middle) and sterile apyrogenic PBS (top, right) as negative control.

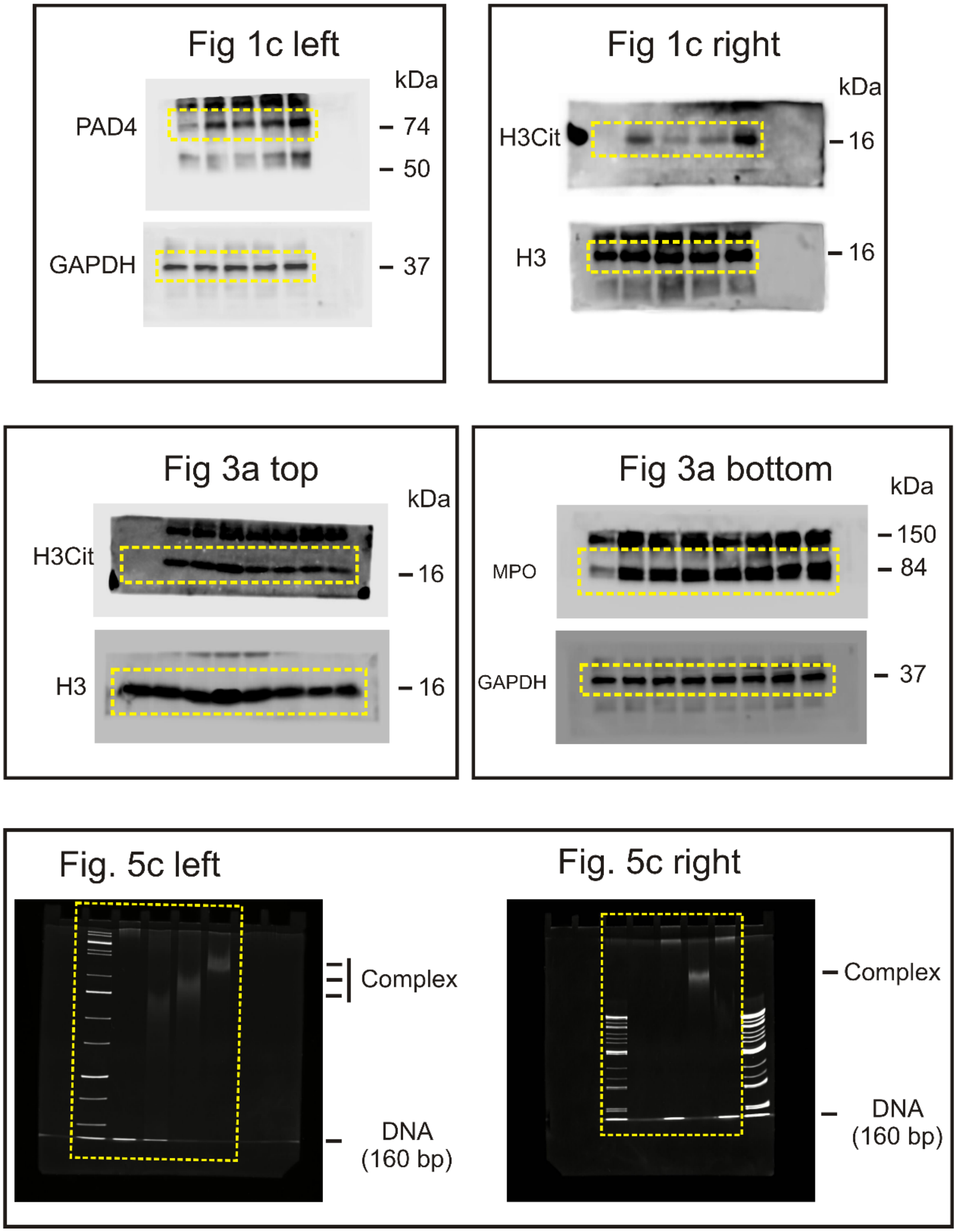

Supplementary Figure 11: Uncropped Western blots and gels

Supplementary Figure 11 continue

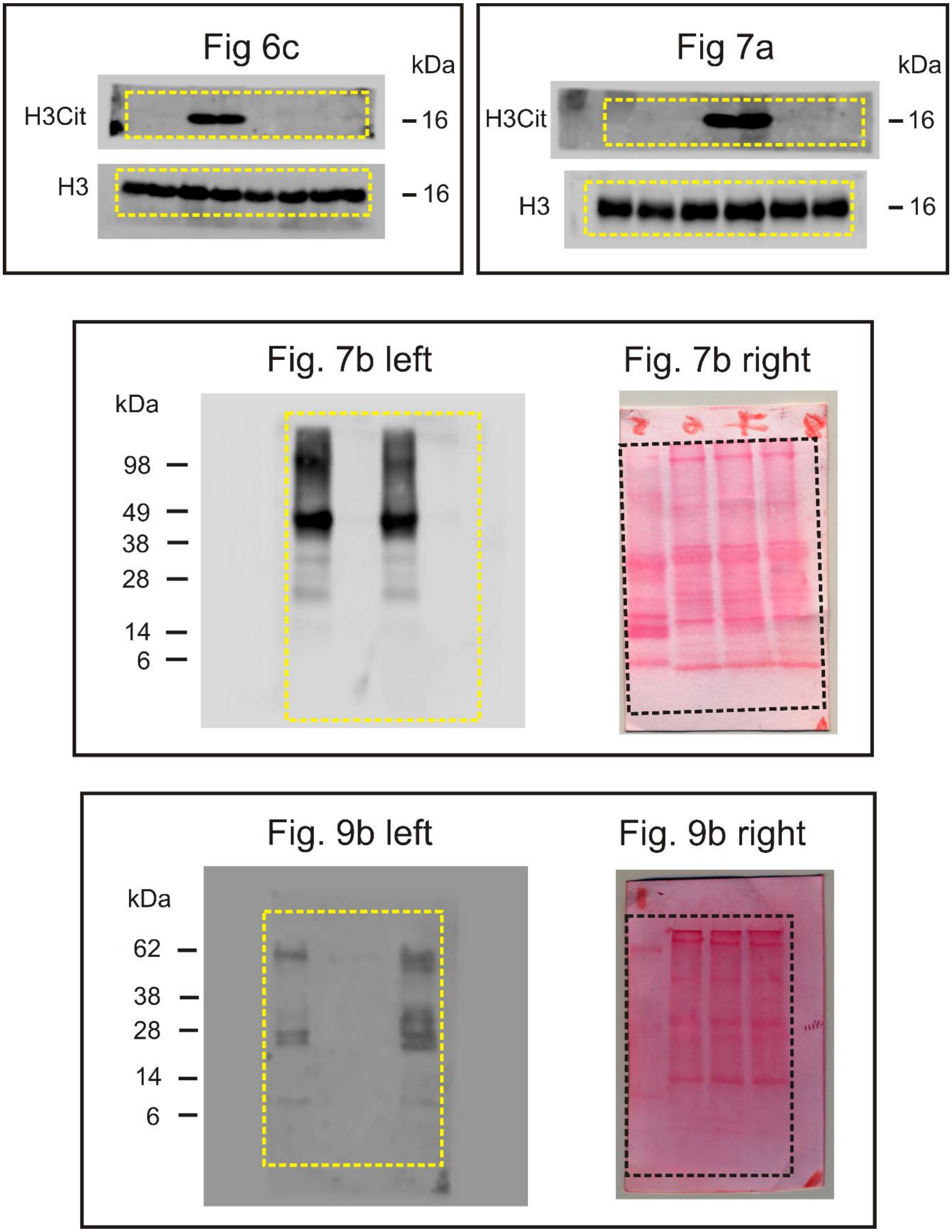

Supplementary Figure 11 continue

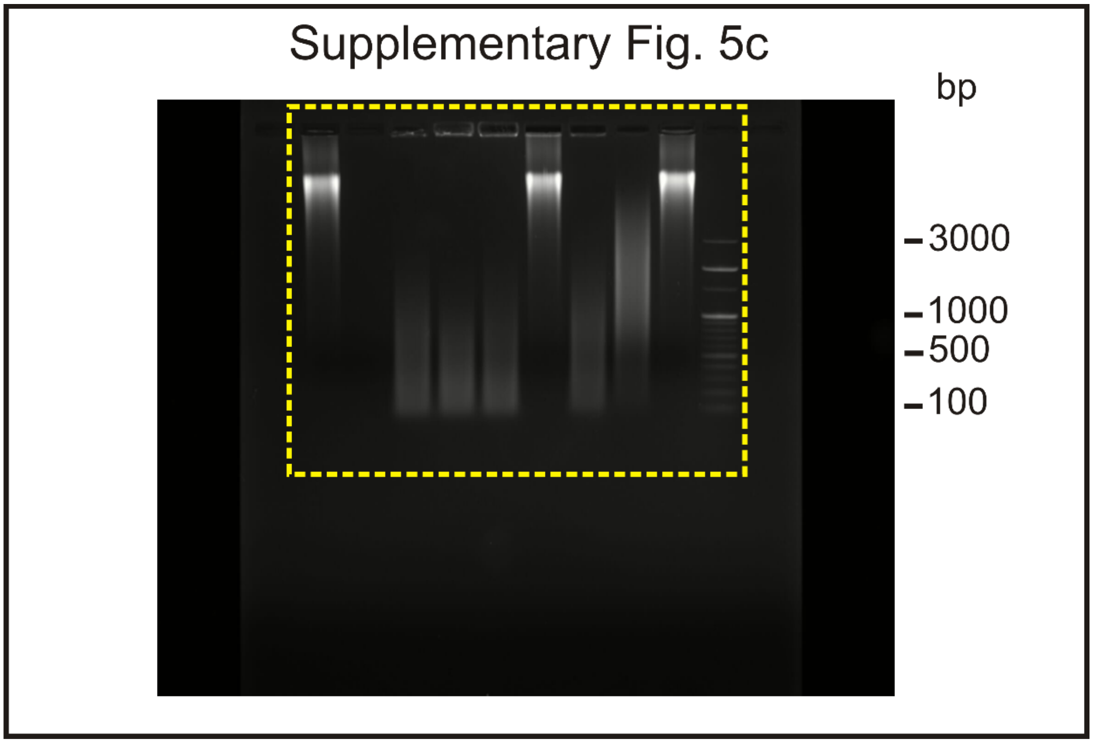

Supplementary Table 1: Differential blood counts of Swiss albino female mice

Day after 1st dose of cyclophosphamide	Total leukocytes (cells mm^{-3}) ± S.E.M	Neutrophils (cells mm^{-3}) ± S.E.M	Lymphocytes (cells mm^{-3}) ± S.E.M	Monocytes (cells mm^{-3}) ± S.E.M
Day 4	950 ± 65	0	143 ± 55	20 ± 8
Day 5	154 ± 39	0	90 ± 35	19 ± 10
Normal range	2500 - 6900	230 - 3500	657 - 5500	25 - 500

Total number of leukocytes and differential count in peripheral blood of cyclophosphamide injected mice (n=10) and control mice (n=6) were assessed at day 4 and 5.
